# A non-human primate combinatorial system for long-distance communication

**DOI:** 10.1016/j.isci.2024.111172

**Published:** 2024-10-15

**Authors:** Quentin Gallot, Cassandre Depriester, Steven Moran, Klaus Zuberbühler

**Affiliations:** 1 Institute of Biology, University of Neuchâtel, Neuchâtel, Switzerland; 2 Taï Monkey Project, Centre Suisse de Recherches Scientifiques en Côte d'Ivoire, Taï, Cote d'Ivoire; 3 ENES Bioacoustics Research Laboratory, CRNL, University of Saint-Etienne, Saint-Etienne, France; 4 Department of Anthropology, University of Miami, Coral Gables, FL, USA; 5 School of Psychology and Neuroscience, University of St Andrews, St Andrews, UK

**Keywords:** Biological sciences, Behavioral neuroscience, Social sciences, Linguistics

## Abstract

Complex vocal systems are thought to evolve if individuals are regularly challenged by complex social decision-making, the social complexity hypothesis. We tested this idea on a West African forest non-human primate, the Olive colobus monkey, a highly cryptic species with very little social behavior and very small group sizes, factors unlikely to favor the evolution of complex communication. The species also has an unusual fission-fusion social system, with group members regularly spending considerable amounts of time with neighboring groups. As predicted by the social complexity hypothesis, we only found a very basic repertoire of two call types in this species, produced by both males and females. However, the calls were astonishingly loud, never uttered alone but in syntactically structured sequences assembled along a set of rules. We concluded that the Olive colobus monkeys have evolved a combinatorial system to interact with distant group members.

## Highlights

• Olive colobus monkeys produce 2 call types, the most basic repertoire known to date
• Both sexes call in sequences which travel far to neighboring individuals
• Sequences have syntactic structure following several rules
• Some call combinations are strongly referential to different danger types

## Graphical abstract

**Figure:**
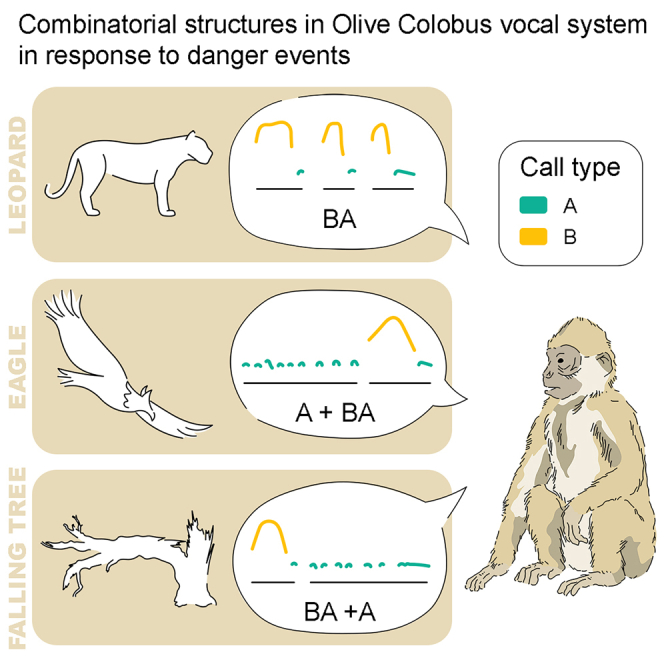

## Introduction

Humans are unique in a number of ways, but perhaps most clearly in their ability to communicate with language, so how did this capacity evolve from earlier forms of communication? A pioneering attempt to address this question was by Hockett,^1^ who proposed a number of design features of human language and noted that most were shared with animal communication systems, with only a few being unique to human language. One such feature is double articulation, or duality of patterning,^2^ evidenced by the fact that all languages combine acoustic components at two distinct levels, phonemes into morphemes and morphemes into words, which gives language its unique generative power. As a result, language is compositional, capable of generating new meanings by combining already meaningful units according to rules.^3^^,^^4^ This multi-layered generative nature of language appears to be unique with no known animal analogue, setting humans apart from all other species (see in the studies by de Boer B et al., Ladd et al., and Roberts et al. ^5^^,^^6^^,^^7^ for discussion).

Composition requires combination, so what are the evolutionary precursors of combinatorial signaling in human language? The last two decades have seen an explosion of studies showing combinatorial capacities in the vocal communication systems of many avian and mammalian species.^8^^,^^9^^,^^10^^,^^11^ Considerable effort has been devoted to animal song, with well-documented examples of highly complex combinations, but no clear evidence of compositionality. As a general pattern, animal song rarely refers to external events, but functions to highlight the quality of the caller or to facilitate social bonding (e.g., humpback whales, *Megaptera novaeangliae*^12^). An interesting exception is the song system of lar gibbons (*Hylobates lar*), which combines meaningless song units into meaningful song types that function in referring to different external events.^13^^,^^14^

Apart from songs, there is some evidence for meaningful call combinations in vocal behavior. Occasionally, such call combinations even seem to be compositional because the overall meaning of the structures derives from the meaning of the constituent parts (^15^, but see in the study by Schlenker et al.^16^). A recent example is chimpanzees (*Pan troglodytes*) producing two context-specific call types, “alarm-huus” (when surprised) and “waa-barks” (when recruiting others). The two calls can be produced on their own or combined into sequences, i.e., “alarm-huu + waa-bark”, which carries an altered meaning derived from the meaning of its components.^17^

An alternative interpretation to animal call compositions is the notion of idiomatic function, which appears to explain the “pyow-hack” combinations of putty-nosed monkeys (*Cercopithecus nictitans*). Here, males produce two types of alarms: “pyow” series to terrestrial disturbances and “hack” series to African crowned eagles (*Stephanoaetus coronatus)*.^18^^,^^19^ Additionally, males sometimes produce mixed sequences, i.e., “pyows” followed by “hacks”, which reliably predict group movement regardless of the external referent that originally triggered to calling behavior.^20^^,^^21^ Another example is the alarm call system of black-fronted titi monkeys (*Callicebus nigrifrons*) where individuals produce two alarm calls, “A” and “B”, which can be combined into long sequences. When encountering unusual danger event (e.g., terrestrial predators in the canopy or aerial predators on the ground), titi monkeys produce mixed sequences with “A” and “B” calls that contain information about both the type and location of predator, encoded probabilistically with meaning related to the proportion of call bigrams (i.e., two contiguous calls among all the contiguous pairs of calls of the sequence).^22^^,^^23^ Note that there are alternative explanations, such as the “urgency” principle proposed by Schlenker et al.,^24^ to explain the relationship between structure and meaning.

There is currently no coherent theoretical framework that could explain the diversity of animal signal combinations.^11^ Regarding the emergence of communicative complexity, the lead hypothesis is that vocal complexity follows social complexity, usually when individuals live in complex social structures that require them to constantly navigate between cooperation and competition (the social complexity hypothesis, ^25^^,^^26^). Alternative theories for the emergence of complex communication are rooted in the impact of high predation pressure^27^ or needs in relation to long-term parental care.^28^

In this study, we investigate the vocal communication system of Olive colobus monkeys (*Procolobus verus*), a forest-living African primate that forms small but fluid social groups of 2–15 individuals.^29^ The species engages in little active social behavior^30^ and generally leads a highly cryptic lifestyle, supported by a very cryptic physical appearance. In Taï Forest, Olive colobus monkeys are permanently associated with sympatric monkey species, mostly Diana monkeys (*Cercopithecus diana*;^29^) but also other non-human primate species. Their social system is very unusual, with individuals occasionally visiting neighboring groups, with transits taking place when the two host groups are near each other.^31^ Both males and females vocalize, but only rarely and with an extremely basic repertoire of two call types.^32^^,^^33^ However, several researchers have reported that, if individuals call, they do so in long sequences and that these sequences travel far beyond their own group through dense forest habitat.^32^^,^^33^^,^^34^^,^^35^ The goal of our study was to investigate the structure of the vocal sequences produced in different circumstances. To this end, we first established the species’ call and sequence repertoire to then determine the combinatorial rules.

## Results

### Call repertoire

We inspected nearly 6 h of cumulative recordings (357 min) with a total of *N* = 1,246 calls, consisting of systematically elicited vocal responses to different types of dangers. Recordings were extracted from three different datasets, all collected from unhabituated Olive colobus groups. Calls from datasets 1 and 2 were in response to playbacks of predator vocalizations (leopard growls *Panthera pardus*, eagle shrieks, chimpanzee pant-hoots) and sounds of falling trees, while data from dataset 3 consisted of calls recorded in response to visual models of a leopard and a crowned eagle (*N* = 119 trials with vocal responses; see STAR Methods for details on dataset 1: *N* = 18 trials; dataset 2: *N* = 91 trials; dataset 3: *N* = 10; Table 1).

**Table 1:** Olive colobus dataset composition

Dataset	Description	Experimenter	N trial with response	Total N trials	Year	Approx. N groups a
1	predator playbacks	KZ	18	24	1994–99	11
2	predator and tree playbacks	QG	70	157	2021–22	16
	tree playbacks	CD	21	44	2022	15
3	predator models	QG	10	25	2022	8

a Based on the spatial estimates.

**Figure 1:**
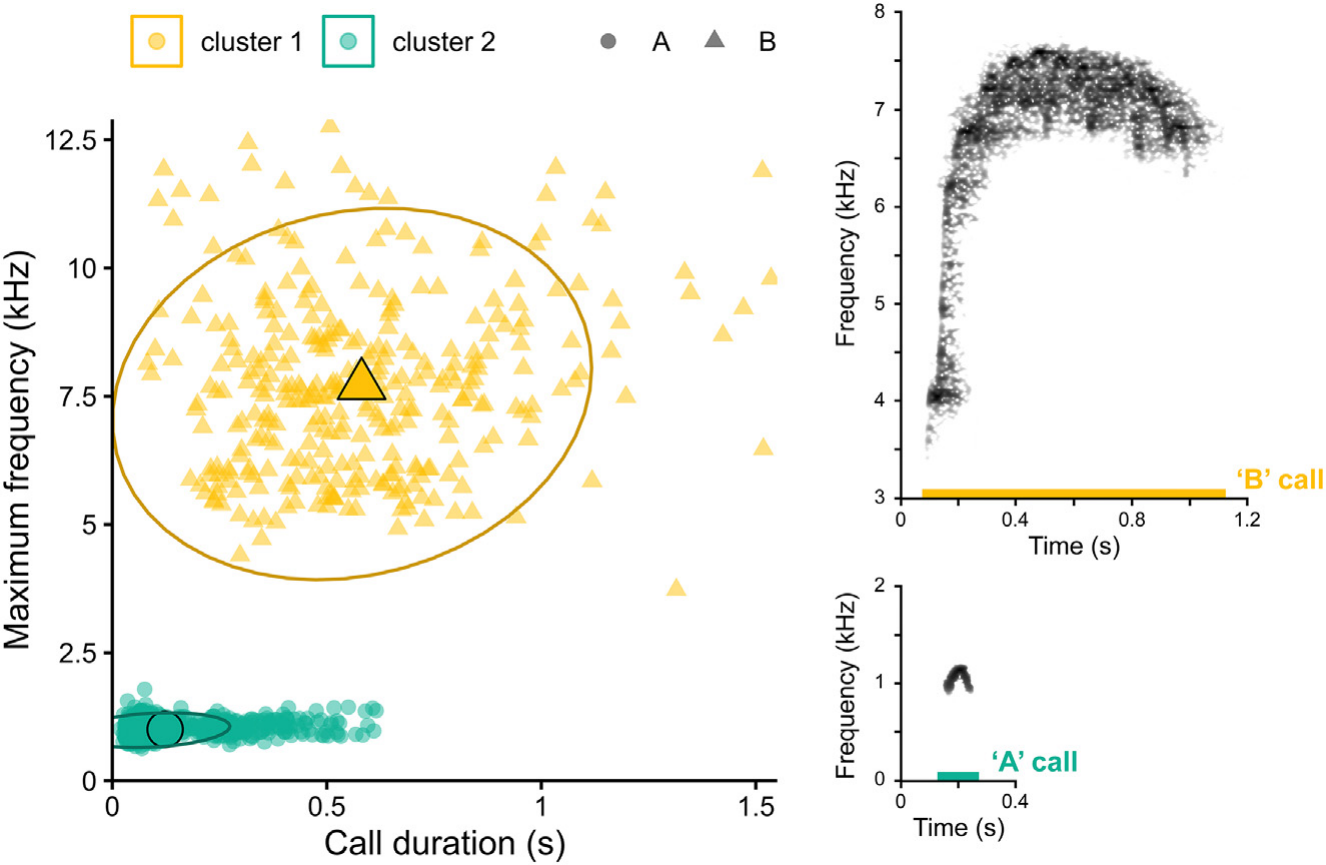
Results from Olive colobus monkey call classification plotted in acoustic parameter space with corresponding spectrograms Each data point represents one call (circles = “A” calls, triangles = “B” calls). Colors represent the cluster attributions from the Partitioning Around Medoids analysis. Each ellipse was determined using the Student’s t-distribution with 95% of confidence level; center dots represent the mean position of the data points for each cluster. Spectrograms were extracted from Raven Pro software v1.6.4,^36^ background noise removed with Adobe Photoshop software v23.0.0.^37^

Confirming pilot observations, the calls could be categorized into two groups, using unsupervised cluster analysis (Partitioning Around Medoids method, Figure S1) with 100% of calls correctly classified (Figure 1): a short, low-frequency call “A” (maximum frequency = 1.00 ± 0.16 kHz (SD); duration = 0.12 ± 0.12 s (SD); *N* = 921) and a long, high-frequency call “B” (maximum frequency = 7.72 ± 1.73 kHz (SD); duration = 0.58 ± 0.28 s (SD); *N* = 325). Both males and females produced both call types (Figure S2). The calls were relatively loud and audible over considerable distances, much beyond the immediate group.

### Response rates

For datasets 2 and 3 (see STAR Methods), it was possible to estimate the response rates of Olive colobus monkeys to danger-related stimuli (i.e., the percentage of trials in which at least one Olive colobus from inside the group vocalized within the first 160 s of recording). For the playback stimuli (dataset 2), the response rates were as follows: leopard: 61.4% (*N* = 44 trials); eagle: 36.7% (*N* = 60 trials); falling tree: 50.6% (*N* = 83 trials); chimpanzee: 0.0% (*N* = 14 trials). For the visual stimuli (dataset 3), only leopard models elicited vocal responses: leopard: 66.7% (*N* = 15 trials); eagle: 0.0% (*N* = 10 trials).

### Syntax

**Figure 2:**
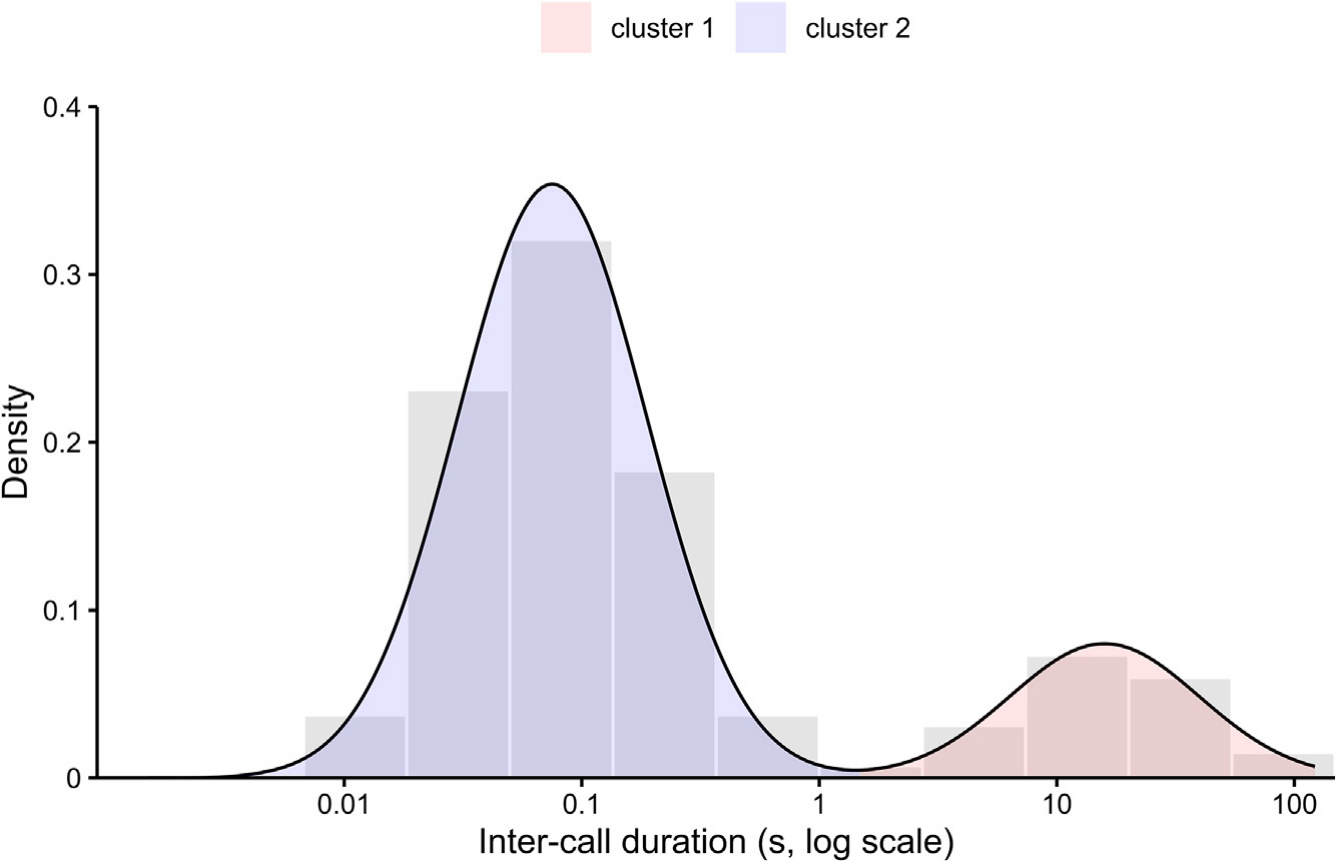
Density histogram and kernel density estimate curve of Olive colobus inter-call intervals The x axis was log transformed. The blue and red clusters were obtained via unsupervised model-based clustering and represent the distributions of inter-call intervals (*N* = 1,119) within and between sequences, respectively.

Olive colobus monkeys almost always produced sequences of calls in different combinations. In a first analysis, we segmented the utterances into discrete sequences by analyzing the distribution of the inter-call intervals. We were able to measure *N* = 1,119 inter-call intervals across the three datasets, which revealed a multimodal distribution. We then conducted an unsupervised model-based clustering, which returned a distribution with two modes as the optimal fit for the data with no overlap (bootstrap likelihood ratio test: *N* = 1,000 iterations; 1 vs. 2 modes: χ^2^ = 963.511, *p* < 0.001; 2 vs. 3 modes: χ^2^ = 0.477, *p* = 0.166; Figure 2). The separation occurred for inter-call intervals between 1 and 2 s, which then led us to the definition of a call sequence as a series of at least two calls, separated by inter-call intervals of less than 1 s (mean = 0.12 ± 0.14 s SD). Between-sequence intervals lasted on average 22.8 ± 21.3 s (SD).

**Figure 3:**
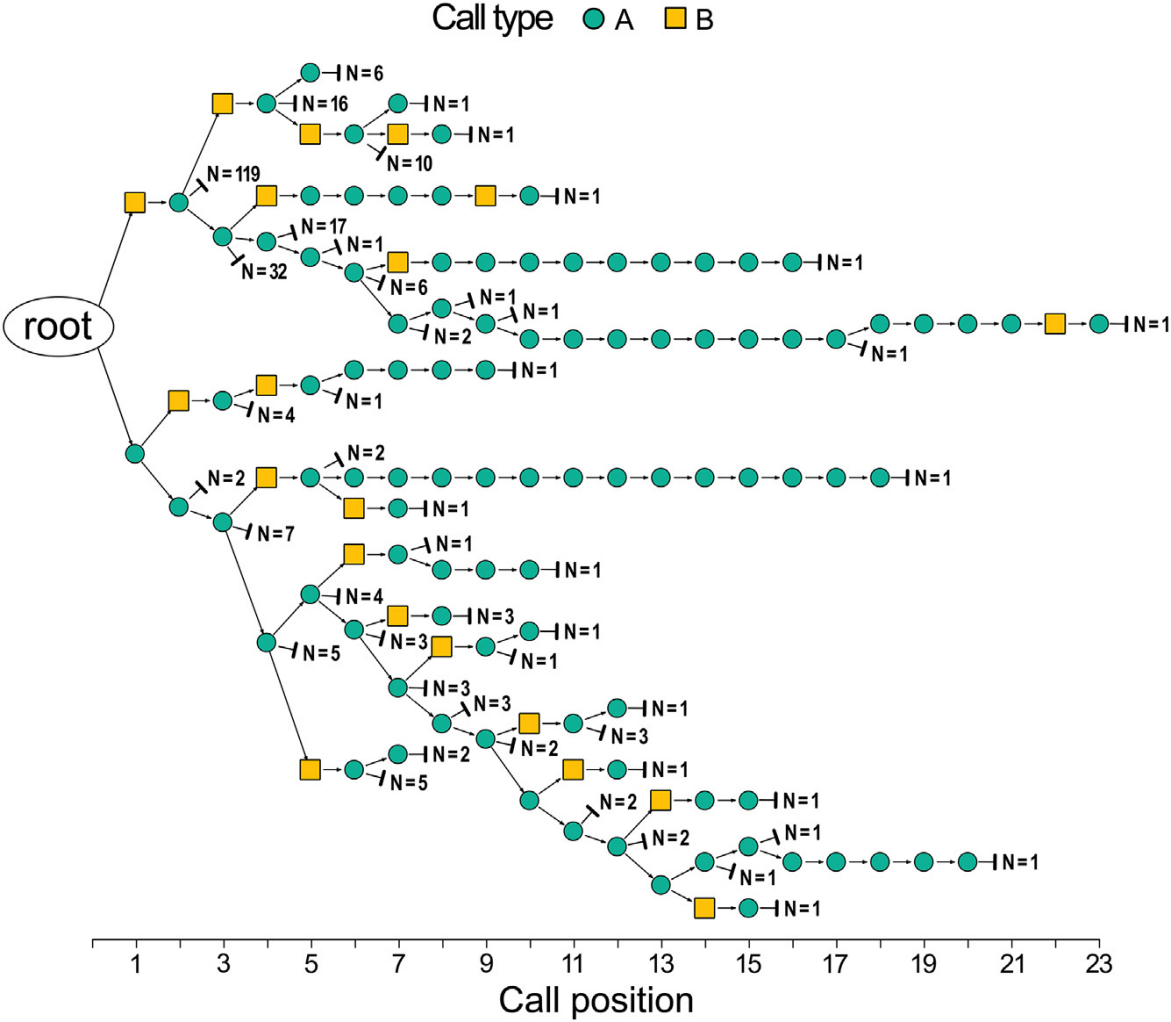
Trie of *N* = 284 call sequences grouped into 48 sequence types in response to different disturbances: leopard and eagle calls presented from the ground or within a tree, falling tree sounds, and visual leopard model Each colored node represents a call (green = “A”; yellow = “B”). The “root” node represents the start and “⊣” marks the end of a sequence. The number at the end of each branch represents the sample size of the corresponding sequence in all datasets. The x-coordinates represent the ordinal position of the call in the sequence.

In all datasets, if vocal responses occurred, they were only produced by one individual of the group (male or female) and practically always as a sequence of calls (96.5%; *N* = 1,246 calls), with a mean sequence length of 4.2 ± 3.3 calls (SD) and a maximum of 23 calls. We identified 48 different sequence types, 24 of which (50.0%) were only recorded once (Figure S3). Most sequences were combinations of both call types (87.3%; *N* = 284 sequences); the rest were repetitions of “A” calls (12.7%). Repetitions of “B” calls were never recorded (0.0%). We then computed a “trie”, using all *N* = 284 sequences (Figure 3) and identified the following set of rules.

Using mathematical denotation, we identified three rules, by letting $n_{i}$ denote the number of components of the i-th sequence, with i = 1, …, N and $S_{i,k}$ be the value taken by the k-th component of the i-th sequence with k = 1, …, $n_{i}$ and i = 1, …, *N*. The values were either equal to A or B. I was used as an indicator variable.

(1) *A-Dominance*: the number of “B” calls never exceeds the number of “A” calls, i.e., in a sequence $S_{i}$ , twice the sum from k = 1 to $n_{i}$ of the values equal to A is always greater than $n_{i}$:

$$\text{if}\;I\left(S_{i,k}=A\right)=\left\{ \begin{array}{c} 1\;\text{if}\;S_{i,k}=A\\ 0\;\text{otherwise} \end{array}\right.,\text{then}\;2\sum_{k=1}^{n_{i}}I\left(S_{i,k}=A\right)>n_{i},\forall \;i\in \left\{1,...,N\right\}$$

(2) *A-Suffixation*: sequences always end with “A” calls, i.e., the value at the terminal position (k = $n_{i}$) in a sequence $S_{i}$ is always equal to A:

$$\text{if}\;k=n_{i},\text{then}\;S_{i,k}=A,\forall \;i\in \left\{1,...,N\right\}$$

(3) *B-Singularity:* “B” calls are never repeated twice in succession, i.e., in a sequence $S_{i}$, if the value at position k is equal to B for all k between 1 and $n_{i}$-1, then the value at the next position k +1 is always A:

$$\text{if}\;S_{i,k}=B,\text{then}\;S_{i,k+1}=A,\forall \;i\in \left\{1,...,N\right\},k\in \left\{1,...,n_{i}-1\right\}\;\text{.}$$

**Figure 4:**
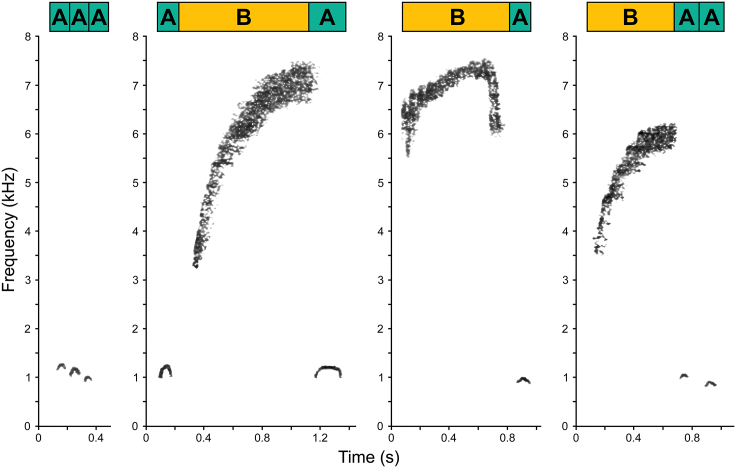
Spectrographic representations of the four basic sequence types to different types of dangers “AAA” and “ABA” sequences shown here were given in response to eagle shrieks, the “BA” sequence was given in response to a leopard growl and the “BAA” sequence was given in response to a falling tree sound. Spectrograms were extracted from Raven Pro software v1.6.4,^36^ background noise removed with Adobe Photoshop software v23.0.0.^37^

Figure 3 summarizes all *N* = 284 sequences recorded over eight years of research. 96.1% of them belong to four basic sequence types: (1) type “A” i.e., *N* = 36 repetitions of “A calls”; (2) type “BA” i.e., *N* = 146 “BA-gram(s)”; (3) type “A + BA” i.e., *N* = 23 “A” call(s) followed by “BA-gram(s)”, and (4) type “BA + A” i.e., *N* = 68 “BA-gram(s)” followed by “A” call(s) (see Figure 4 for spectrographic representations; Figure S4 for spectrograms of master recordings; Table S1 for detail on *N* = 11 unclassified sequences).

### Sequence initiation

**Figure 5:**
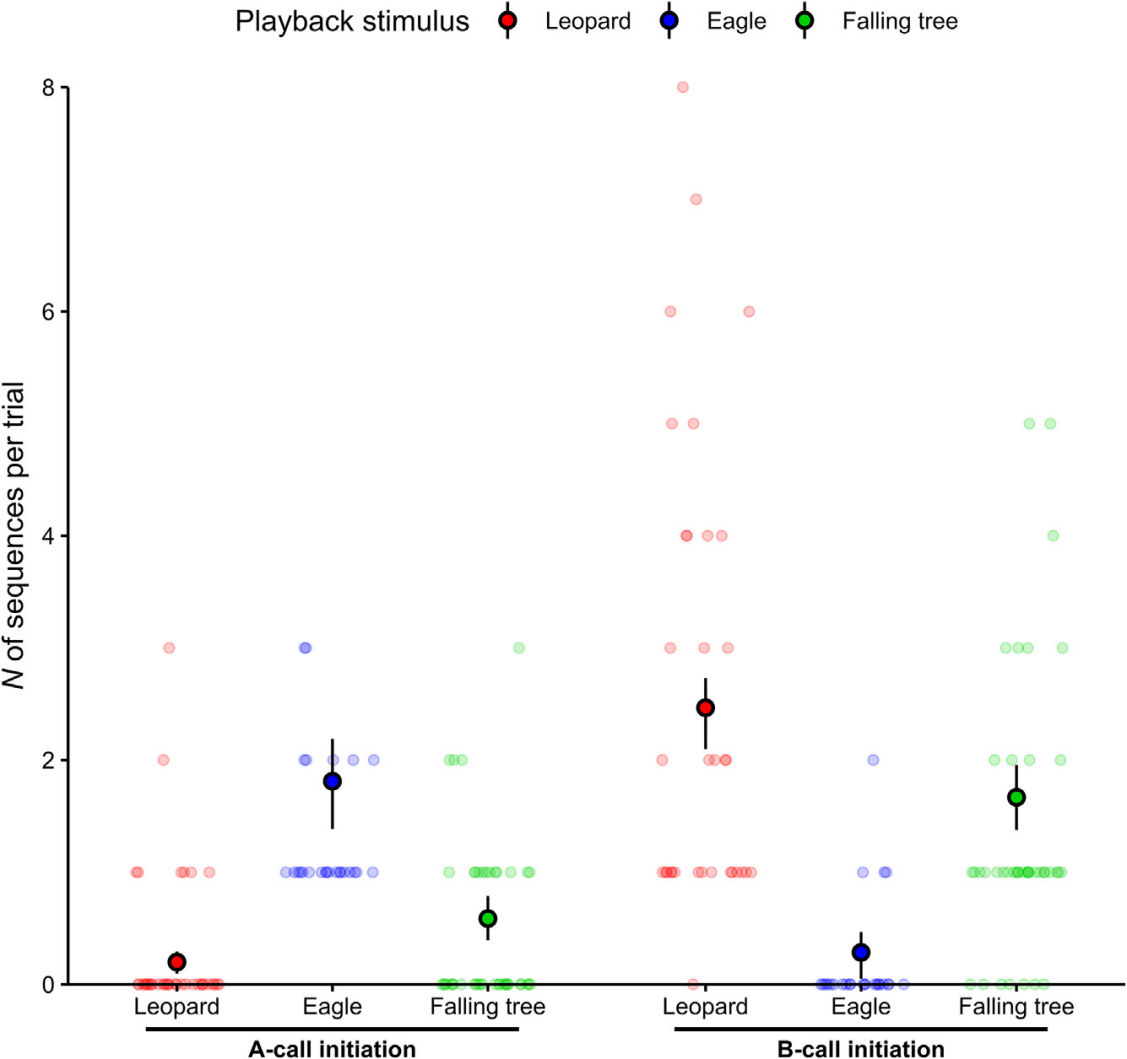
Context-specificity of sequence initiation in Olive colobus call production Each data point represents one trial, and vocal production from only one individual of the group. To reduce overplotting and enhance visibility, random noise was added to the x-coordinate of data points. Embedded black dots and vertical lines indicate means and bootstrapped 95% confidence intervals from model estimation, respectively. Sample sizes: Leopard growls *N* = 37 trials, Eagle shrieks *N* = 25 trials, Falling tree sounds *N* = 42 trials. Three outliers not depicted (*N* = 2 leopard trails with 13 “B”-initiated sequences, and *N* = 1 leopard trial with 9 “B”-initiated sequences).

Sequences could start with either call type (“A” or “B”), which appeared to determine whether or not an eagle was the cause. Overall, if focusing on the sequence’s first call, there was a clear effect of the playback stimulus on the quantity of the different sequence types produced (*p* < 0.001, refitted GLMM1, Table 2; Figure 5, dataset 1 and 2).

**Table 2:** Results of GLMM1 exploring the effect of playback stimulus type and group location on the number of sequences starting with call A or call B (*N* = 104 trials)

Sequence initiation								
GLMM1 refit formula^a^: N_sequences ∼ call_type * stimulus + group_location + offset(log(N_tot_sequences +1)) + (1 | trial_ID)								
Predictors	Estimate	SE	95% CI	min	max	χ^2^	df	p
(Intercept)	−0.644	0.142	[-0.968, −0.392]	−0.723	−0.602	–	–	b
call_type^c^ *B*	– −1.849	– 0.425	– [-2.963, −1.170]	– −2.354	– −1.743	– –	– –	b –
stimulus^d^ *leopard* *tree*	– −2.207 −1.127	– 0.286 0.211	– [-2.894, −1.691] [-1.594, −0.737]	– −2.494 −1.269	– −2.099 −1.057	– – –	– – –	b – –
group_location^e^ *south*	– 0.019	– 0.105	– [-0.189, 0.235]	– 0.000	– 0.117	0.033 –	1 –	0.857 –
call_type: stimulus *B: leopard* *B: tree*	– 4.366 2.893	– 0.501 0.468	– [3.528, 5.735] [2.125, 4.249]	– 4.229 2.779	– 4.877 3.399	124.898 – –	2 – –	**<0.001** – –

The model was refitted without non-significant predictor interactions. CI refers to the bootstrapped confidence interval (*N* = 1,000 iterations) and min and max to minimum and maximum estimate from stability analysis. Significant results are highlighted in bold.

a Testing main effect of predictors after removing non-significant interaction from the model; GLMM1, call_type: group_location, χ^2^ = 0.759, df = 1, *p* = 0.384.

b Not depicted because of limited interpretability.

c Estimate refer to comparison with reference category “A”.

d Estimates refer to comparison with reference category “eagle”.

e Estimate refer to comparison with reference category “north”.

Responses to eagle shrieks consisted almost exclusively of sequences starting with an “A” call (eagle-ground 84.8% of *N* = 33 sequences; eagle-tree 100% of *N* = 6 sequences). Olive colobus monkeys also produced significantly more sequences with “A” initiation to eagles than any other danger (pairwise post-hoc comparisons on refitted GLMM1: Table S2; Figure 5). In addition, both responses to leopard growls and falling tree sounds were made from a majority of sequences starting with a “B” call (leopard-ground 90.3% of *N* = 113 sequences; leopard-tree 100% of *N* = 20 sequences; falling tree 73.4% of *N* = 79 sequences). Monkeys produced significantly more sequences with “B” initiation to leopards and falling trees than to eagles (pairwise post-hoc comparisons on refitted GLMM1: Table S2; Figure 5). This specificity in sequence production (“A” to eagle, “B” to leopard) was independent of the elevation of the danger, i.e., whether the sounds were played from the ground or within a tree.

Neither group location (*p* = 0.857, refitted GLMM1, Table 2) nor playback elevation (*p* = 0.119, trials for leopard and eagle playbacks only, refitted GLMM2, Table S3) had a significant effect on the quantity of the different sequence types produced during a trial, neither in interaction with other predictors or alone. Notably, this was also independent of mode, i.e., visual or sound stimulus, with responses to leopard visual model (dataset 3) being similar to responses to leopard playbacks: 97.0% (*N* = 33) of sequences started with a “B” call.

### Sequence types

**Figure 6:**
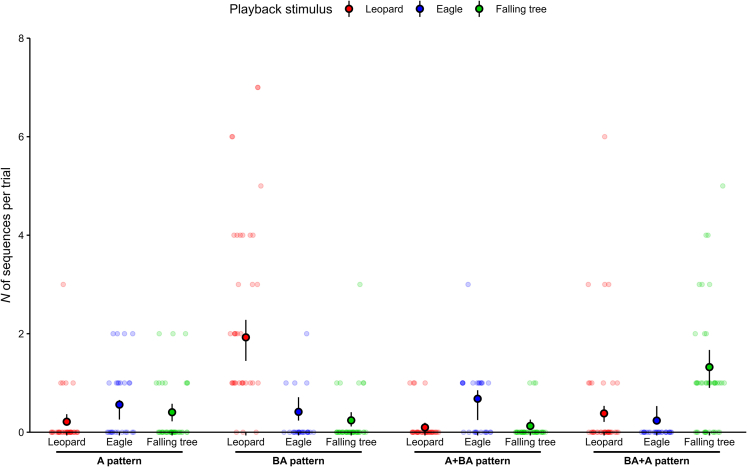
Context-specificity of overall sequence patterns in Olive colobus call production Each data point represents one trial, and vocal production from only one individual of the group. To reduce overplotting and enhance visibility, random noise was added to the x-coordinate of each data point. Embedded black dots and vertical lines indicate means and bootstrapped 95% confidence intervals from model estimation, respectively. Sample sizes: Leopard growls *N* = 37 trials, Eagle shrieks *N* = 25 trials, Falling tree sounds *N* = 42 trials. One outlier not depicted (*N* = 1 leopard trail with 12 “BA” sequences).

Out of the four sequence types recorded in response to playbacks (dataset 1 and 2), three of them reliably distinguished the danger type. If focusing on the overall sequence pattern, there was a clear effect of the playback stimulus on the quantity of the different sequence types produced during a trial (*p* < 0.001, refitted GLMM3, Table 3; Figure 6).

**Table 3:** Results of GLMM3 exploring the effect of playback stimulus type and group location on the number of sequences for each sequence pattern (*N* = 104 trials)

Sequence pattern								
GLMM3 refit formula^a^: N_sequences ∼ pattern * stimulus + group_location + offset(log(N_tot_sequences +1)) + (1 | trial_ID)								
Predictors	Estimate	SE	95% CI	min	max	χ^2^	df	p
(Intercept)	−1.767	0.273	[-2.553, −1.549]	−1.841	−1.729	–	–	b
pattern^c^ *BA* *A + BA* *BA + A*	– −0.304 0.193 −0.862	– 0.361 0.329 0.405	– [-0.505, 0.616] [-0.487, 0.819] [-1.656, 0.160]	– −0.480 0.017 −0.915	– −0.202 0.313 −0.772	– – – –	– – – –	b – – –
stimulus^d^ *leopard* *tree*	– −0.964 −0.323	– 0.325 0.313	– [-1.066, −0.079] [-0.618, 0.400]	– −1.138 −0.408	– −0.903 −0.261	– – –	– – –	b – –
group_location^e^ *south*	– −0.016	– 0.162	– [-0.308, 0.351]	– −0.029	– 0.041	0.010 –	1 –	0.922 –
pattern: stimulus *BA: leopard* *A + BA: leopard* *BA + A: leopard* *BA: tree* *A + BA: tree* *BA + A: tree*	– 2.503 −0.979 1.443 −0.215 −1.348 2.043	– 0.4205 0.522 0.481 0.489 0.532 0.457	– [1.679, 3.327] [-1.816, −0.251] [0.008, 1.444] [-1.249, 0.201] [-1.853, −0.364] [1.148, 2.937]	– 2.407 −1.134 1.178 −0.541 −1.525 1.953	– 2.687 −0.820 1.616 −0.067 −1.215 2.138	119.170 – – – – – –	6 – – – – – –	**<0.001** – – – – – –

The model was refitted without non-significant predictor interactions. CI refers to the bootstrapped confidence interval (*N* = 1,000 iterations), min and max to minimum and maximum estimate from stability analysis. Significant results are highlighted in bold.

a Testing main effect of predictors after removing non-significant interaction from the model; GLMM3, pattern: group_location, χ^2^ = 0.171, df = 3, *p* = 0.982.

b Not depicted because of limited interpretability.

c Estimates refer to comparison with reference category “A”.

d Estimates refer to comparison with reference category “eagle”.

e Estimate refer to comparison with reference category “north”.

Responses to leopards were made from “BA” sequences (leopard-ground 71.7% of *N* = 113 sequences; leopard-tree 100% of *N* = 20 sequences); responses to eagles consisted of a majority of “A + BA” sequences (eagle-ground 36.4% of *N* = 33 sequences; eagle-tree 50.0% of *N* = 6 sequences); and responses to falling trees were made of “BA + A” sequences (59.5% of *N* = 79 sequences). Context specificity was also present for “BA” sequences, significantly more often produced to leopard growls than other danger types, for “A + BA” sequences to eagle shrieks, and for “BA + A” sequences to falling trees (pairwise post-hoc comparisons on refitted GLMM3: Table S4; Figure 6). “A” sequence was the only sequence pattern that could not be associated with a single danger type (pairwise post-hoc comparisons on refitted GLMM3: Table S4; Figure 6).

If considering all playback trials (i.e., first 160 s of recordings, see STAR Methods), callers were very consistent in the type of sequences they produced to the different dangers, with unique sequences produced in 71.7% of trials (*N* = 113; all datasets).

As before, we found no effect of group location or whether callers responded to leopard growls or eagle shrieks presented from the ground or a tree. In interaction with other predictors or alone, neither group location (*p* = 0.922, refitted GLMM3, Table 3) nor playback elevation (*p* = 0.241, trials for leopard and eagle playbacks only; refitted GLMM4, Table S5) had a significant effect. Again, this was independent of the stimulus mode, i.e., visual or sound stimulus, with responses to leopard visual model (dataset 3) being similar to responses to leopard playbacks: 97.0% (*N* = 33) of sequences from “BA” type.

### Sequence termination

**Figure 7:**
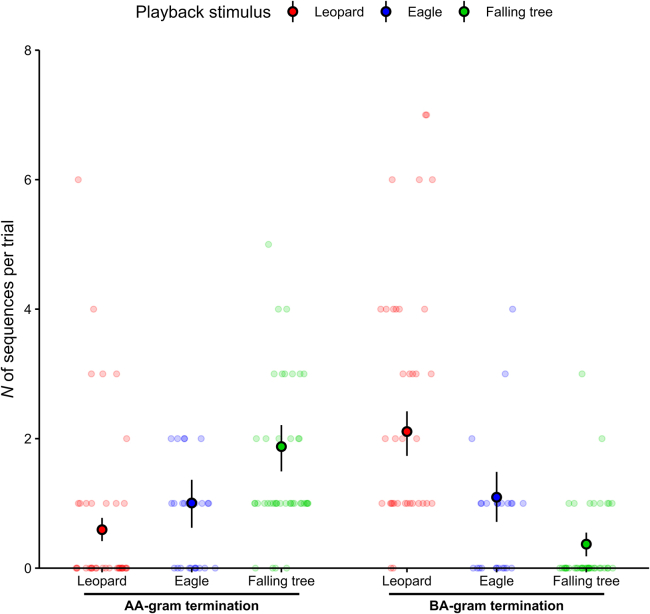
Context-specificity of sequence termination in Olive colobus call production Each data point represents one trial, and vocal production from only one individual of the group. To reduce overplotting and enhance visibility, random noise was added to the x-coordinate of each data point. Embedded black dots and vertical lines indicate means and bootstrapped 95% confidence intervals from model estimation, respectively. Sample sizes: Leopard growls *N* = 37 trials, Eagle shrieks *N* = 25 trials, Falling tree sounds *N* = 42 trials, one outlier not depicted (*N* = 1 leopard trail with 12 “BA-gram” terminated sequences).

Sequences always terminated with either an “AA-gram” or a “BA-gram” (45.0% or 55.0%, respectively; *N* = 251; dataset 1 and 2), but never with “AB-gram” or “BB-gram”. Overall, if focusing on the sequence’s last bigram, there was a clear effect of the playback stimulus on the quantity of the different sequence types produced during a trial (*p* < 0.001, refitted GLMM5, Table 4; Figure 7, dataset 1 and 2).

**Table 4:** Results of GLMM5 exploring the effect of playback stimulus type and group location on the number of sequences ending with AA-gram or BA-gram (*N* = 104 trials)

Sequence termination								
GLMM5 refit formula^a^: N_sequences ∼ bigram_type * stimulus + group_location + offset(log(N_tot_sequences +1)) + (1 | trial_ID)								
Predictors	Estimate	SE	95% CI	min	max	χ^2^	df	p
(Intercept)	−1.232	0.216	[-1.745, −0.875]	−1.298	−1.133	–	–	b
bigram_type^c^ *BA-gram*	– 0.085	– 0.264	– [-0.467, 0.625]	– −0.172	– 0.200	– –	– –	b –
stimulus^d^ *leopard* *tree*	– −0.527 0.624	– 0.243 0.213	– [-0.997, −0.031] [0.250, 1.106]	– −0.708 0.526	– −0.460 0.689	– – –	– – –	b – –
group_location^e^ *south*	– 0.020	– 0.123	– [-0.218, 0.287]	– 0.005	– 0.090	0.027 –	1 –	0.869 –
bigram_type: stimulus *BA-gram: leopard* *BA-gram: tree*	– 1.183 −1.708	– 0.311 0.377	– [0.549, 1.815] [-2.557, −1.018]	– 1.066 −1.979	– 1.434 −1.449	98.230 – –	2 – –	**<0.001** – –

The model was refitted without non-significant predictor interactions. CI refers to the bootstrapped confidence interval (*N* = 1,000 iterations), min and max to minimum and maximum estimate from stability analysis. Significant results are highlighted in bold.

a Testing main effect of predictors after removing non-significant interaction from the model; GLMM5, bigram_type: group_location, χ^2^ = 0.055, df = 1, *p* = 0.815.

b Not depicted because of limited interpretability.

c Estimate refer to comparison with reference category “AA-gram”.

d Estimates refer to comparison with reference category “eagle”.

e Estimate refer to comparison with reference category “north”.

Responses to leopard growls consisted almost exclusively of sequences ending with a “BA-gram” (leopard-ground 75.2% of *N* = 113 sequences; leopard-tree 100% of *N* = 20 sequences); responses to eagle shrieks were made of sequences equally ending with both a “BA-gram” or an “AA-gram” (eagle-ground, 51.5% of *N* = 33 sequences ending with a “BA-gram”; eagle-tree 50.0% of *N* = 6 sequences ending with a “BA-gram”); and responses to falling tree sounds consisted of a majority of sequences ending with an “AA-gram” (falling tree 83.5% of *N* = 79 sequences). Interestingly, while “AA-gram” sequence terminations lacked context-specificity (produced to both eagle shrieks and falling trees, pairwise post-hoc comparisons on refitted GLMM5: Table S6; Figure 7), “BA-gram” sequence terminations were highly indicative of predator presence (pairwise post-hoc comparisons on refitted GLMM5: Table S6; Figure 7), unequivocally distinguishing predatory from non-predatory dangers.

As before, we found no effect of group location or on whether the callers responded to predators on the ground or a tree. In interaction with other predictors or alone, neither group location (*p* = 0.869, refitted GLMM5, Table 4) nor playback elevation (*p* = 0.194, trials for leopard and eagle playbacks only; refitted GLMM6, Table S7) had a significant effect. Again, this was independent of the stimulus mode, i.e., visual or sound stimulus, with responses to leopard visual model (dataset 3) resembling responses to leopard playbacks: 100% (*N* = 33) of sequences ended with a “BA-gram”.

## Discussion

By most accounts, Olive colobus monkeys have unusually simple social lives. They form very small groups with an average of 6.7 members, with very little social activity between individuals and no evidence for complex interactions, such as alliance formation, cooperative breeding, reconciliation or consolation.^30^^,^^38^ Their foraging behavior is equally simplistic, by consuming large amounts of mature foliage that other monkeys cannot process, owing to a specialized multi-chambered stomach.^39^ Groups do not appear to make their own travel decisions, but simply follow other monkey species through the forest, mainly crown-living Diana monkeys.^40^ The species is very successful in avoiding predation, is highly cryptic and remarkably fluid in terms of group composition, with individuals regularly commuting between neighboring groups of Diana monkeys.^31^^,^^32^^,^^41^

In this study, we were interested in the vocal behavior of Olive colobus monkeys, for which the leading evolutionary theory for animal communication, the social complexity hypothesis,^25^^,^^26^ makes clear predictions. Low rates of social behavior and absence of decision-making in relation to daily travel suggest very simplistic vocal behavior. In line with this, we found that both males and females rarely vocalized and, if they did, we could only identify two basic call types—one short, low-frequency “A” call and a second elongated, high-frequency “B” call (Figure 1). A cluster analysis showed no overlap between the two call types, but considerable variations in the acoustic structure of both call types, the topic of a forthcoming study.

The two main calls were almost always given as a part of longer sequences in response to external disturbances, i.e., the presence of predatory leopards or crowned eagles as well as falling trees. When analyzing an unprecedentedly large dataset of experimentally elicited call sequences collected in systematic ways over eight years of research, we were able to identify a number of rules that callers adhered to. We also found strong relations between the resulting call sequences and external events, which rendered the utterances referential, the topic of a more detailed future study. Furthermore, we noticed a gradual unfolding and disambiguation of semantic content, whereby the initial call position distinguished between eagle-related and leopard or treefall events and the sequence termination between leopard-related and tree-related disturbances.

Regarding the link between sequence structure and meaning, we were able to determine a number of direct relations. We found four structures, i.e., repetitions of “A” calls, “BA-gram(s)”, “A” call(s) followed by “BA-gram(s)”, and “BA-gram(s)” followed by “A” call(s). “BA-grams” were found in the main body of the sequence. If preceded by “A” call(s) the caller referred to a crowned eagle; if succeeded by “A” call(s), the caller referred to a falling tree. In the absence of such “A” affixes, however, the caller referred to a leopard. Call affixation has already been described in Campbell’s monkey (*Cercopithecus campbelli*) alarm calls, which can be succeeded by optional “-oo” suffixes, an operation that devalues the specificity of the preceding alarm call from highly specific predator references to unspecific warnings of danger.^42^^,^^43^ Bigram-based semantic communication has also been found in titi monkey alarm calling^22^ and bonobo food calling.^44^ For titi monkeys, bigram production is not linked to referents in a categorical but in a probabilistic manner, expressed by the proportion of “BB-grams” relative to other bigrams in the sequence. Similar to what we found in Olive colobus monkeys, the first call already distinguishes between aerial and terrestrial threats.^22^ In contrast to the titi monkey system, we found no evidence for references about the vertical location of the danger (below or above), similar to what has been reported in Diana monkeys.^45^ In further contrast to titi monkeys, the Olive colobus system has additional layers of complexity, insofar as the core element seems to be the “BA-gram”, which can take optional “A” affix(es), before or after, depending on the referent, which seems to qualify as a semantic permutation.^11^ Here, there is some resemblance to putty-nosed monkey alarm calling, where “pyow-hack” sequences predicted group movement,^18^ whereas pure “pyow” and pure “hack” sequences refer to leopards or crowned eagles, respectively.^20^

How did this call system evolve? The social complexity hypothesis does not explain much of what we report here, as it locates the driver for complexification in the daily social challenges individuals have to solve with communication. We and other researchers have found a distinct lack of social interactions, a low number of (within-group) social relations and no evidence for socially strategic, cooperative or otherwise coordinated group-level activities.^32^^,^^41^^,^^46^^,^^47^^,^^48^ Although Olive colobus arguably score low in all these dimensions, they still have evolved a combinatorial system, implemented by a basic vocal repertoire.

One line of explanation for this seemingly counterintuitive finding is related to the amplitude of the call sequences produced in response to danger, which travel well beyond the immediate group to reach more distant listeners.^32^^,^^33^^,^^34^^,^^35^ As mentioned, Olive colobus monkeys have a very flexible and fluid group composition, with individuals regularly visiting neighboring groups.^31^^,^^32^^,^^41^ In all likelihood, the members of neighboring groups know each other and are probably even related to each other, suggesting that the call sequences function in intergroup communication. It is also important to note that, in the experiments, Olive colobus callers systematically waited until other species’ calling efforts had diminished, an indication that the callers aimed to ensure that their signals were heard by distant recipients.

Olive colobus groups are always found in association with other monkey species, especially Diana monkey groups,^29^^,^^40^ an apparently successful antipredator strategy,^49^ enabled by an almost complete avoidance of niche overlap.^29^ Similar to Diana monkeys, Olive colobus responded with silent cryptic behavior to the simulated presence of chimpanzees, and with predator-specific vocal behavior to crowned eagles and leopards (for Diana monkeys, see in the study by Zuberbühler et al.^50^). Galat-Luong^51^ reported further interesting observations to suggest that Olive colobus adapt not only the travel path of their host species, but often also to how they use the vertical strata. For example, individuals have been seen on the main branches of emergent trees in the company of crown-living Western red colobus monkeys (*Piliocolobus badius*), on the ground in the company of mostly terrestrial sooty mangabeys (*Cercocebus atys*), and in the lower strata in the company of lower canopy dwelling Campbell’s and lesser spot-nosed monkeys (*Cercopithecus petaurista*), possible to benefit from a dilution effect in case of a predator attack.^51^ Whether the Olive colobus monkey adapt their vocal behavior (i.e., variation in acoustic properties of calls, or call combinations) according to the composition of the host group remains unclear, and will need further investigation in future research.

In conclusion, our study shows that the social complexity hypothesis is insufficient to explain the evolution of complex communication in a general way, but that constraints related to long-distance communication can also act as an evolutionary driver. Similar arguments have been made for the singing behavior of lar gibbons, who also use syntactic structure to discriminate references to external events from ordinary duetting.^13^^,^^14^ In sum, despite a basic sociality and good protection from predation, this species has evolved one of the most complex combinatorial systems known to date in non-human primates.

### Limitations of the study

Future research will need to focus on the acoustic gradation within the two basic call types, which has not been addressed here but is likely substantial. Both call types appear to have acoustic variants that may encode further layers of information (see in the studies by Fichtel et al., Furrer et al., Manser et al., Sieving et al.,^52^^,^^53^^,^^54^^,^^55^ for examples in other species). Second, to confirm that the form-meaning patterns described in this study are understood by listeners, playback experiments will be needed. Finally, Olive colobus monkeys occasionally produce call sequences in the absence of external events, but seemingly only when in close proximity to neighboring groups,^46^ again suggesting that individuals seek to communicate with their neighbors. Whether such non-predatory call sequences contain compositions other than the ones presented here will also have to be investigated.

## Resource availability

### Lead contact

Further information and requests for resources and reagents should be directed to and will be fulfilled by the lead contact, Quentin Gallot (quent.gallot@gmail.com).

### Materials availability

This study did not generate new unique reagents.

### Data and code availability

• Original acoustic and environmental data have been deposited at SWISSUbase and are publicly available as of the date of publication. DOIs are listed in the key resources table.
• All original code has been deposited at SWISSUbase and is publicly available as of the date of publication. DOIs are listed in the key resources table.
• Any additional information required to reanalyze the data reported in this paper is available from the lead contact upon request.

## Acknowledgments

We thank Landri Bele, Clémentine Bodin, and Arsene Toh Gueye for assistance in setting up the experiments, and all the other members of the Taï Monkey Project for their support: Ferdinand Ouoro Bele, Ernest Kamy Biohomy, Paterson Kalo, Noël Guy Peho, Sébastien Gbamlin, Edmond Baoue. We thank Anderson Bitty and the staff of the *Centre Suisse de Recherches Scientifiques* (CSRS) for logistic support and the *Office Ivoirien des Parcs et Réserves* (OIPR) for permission to conduct research in Taï National Park. We thank Radu Slobodeanu and Yves Tillé for their advice on statistical analyses and mathematical formulations, respectively. Research was funded by the 10.13039/501100001711Swiss National Science Foundation (Project Grant 310030_185324 to KZ; 10.13039/501100023555NCCR Evolving Language, Agreement #51NF40_180888; and PCEFP1_186841 to SM).

## Author contributions

Conceptualization, Q.G. and K.Z.; methodology, Q.G., K.Z., and C.D.; formal analysis, Q.G.; investigation, Q.G., K.Z., and C.D.; resources, K.Z.; data curation, Q.G.; writing—original draft, Q.G.; writing—review and editing, Q.G., K.Z., and S.M.; visualization, Q.G.; supervision, K.Z. and S.M.

## Declaration of interests

The authors declare no competing interests.

## STAR★Methods

### Key resources table

**Table:** 

REAGENT or RESOURCE	SOURCE	IDENTIFIER
**Deposited data**		

Original data deposited for this study	SWISSUbase	https://doi.org/10.60544/nd6p-dk04

**Experimental models: organisms/strains**		

Olive colobus (*Procolobus verus*)	Taï National Parc, Ivory Coast	N/A

**Software and algorithms**		

Code for model building, evaluation, and plotting	SWISSUbase	https://doi.org/10.60544/nd6p-dk04
R: A language and environment for statistical computing v4.3.1	R Core Team^57^	http://www.r-project.org/
Raven Pro 1.6.4	Raven Pro: Interactive Sound Analysis Software^36^	https://ravensoundsoftware.com/software/raven-pro/
Audacity v2.1.0	Audacity Team^58^	https://www.audacityteam.org/
Adobe Photoshop v23.0.0	Adobe Photoshop^37^	https://www.adobe.com/products/photoshop.html
*DHARMa* R package v0.4.6	Hartig^59^	https://cran.r-project.org/web/packages/DHARMa/index.html
*glmmTMB* R package v1.1.9	Brooks et al.^60^	https://cran.r-project.org/web/packages/glmmTMB/index.html
*emmeans* R package v1.8.5	Lenth^61^	https://cran.r-project.org/web/packages/emmeans/index.html
*cluster* R package v2.1.4	Maechler et al.^62^	https://cran.r-project.org/web/packages/cluster/index.html
*factoextra* R package v1.0.7	Kassambara^63^	https://cran.r-project.org/web/packages/factoextra/index.html
*igraph* R package v1.4.2	Csardi and Nepusz^64^	https://cran.r-project.org/web/packages/igraph/index.html
*mclust* R package v6.1.1	Scrucca et al.^65^	https://cran.r-project.org/web/packages/mclust/index.html
*parameters* R package v0.21.6	Lüdecke et al.^66^	https://cran.r-project.org/web/packages/parameters/index.html

**Other**		

3D printable model of an African crowned eagle (*Stephanoaetus coronatus*)	SWISSUbase	https://doi.org/10.60544/g1sx-4j76

### Experimental model and subject details

Data collection in the Taï National Park was approved by the *Office Ivoirien des Parcs et Réserves* (OIPR). Research authorization and Ethics approval has been given by the *Ministère de l’enseignement supérieur et de la Recherche Scientifique* of Ivory Coast (permit number 010/ME/SRS/DGRI). The data used in this study comprise behavioral observations, obtained using playbacks and predator visual models, and sound recordings of wild, free-ranging male and female Olive colobus monkeys. The precise ages of these individuals were unknown.

### Method details

#### Study site and species

The study was conducted in the Taï National Park (Ivory Coast) about 20 km south-east of the town of Taï in an approximately 70 km^2^ area surrounding the ‘Center de Recherche en Ecologie’ (WGS84: N5° 49.9′, W7° 20.5). We analyzed three different datasets, collected over two long study periods (dataset 1: Klaus Zuberbühler KZ, 1994–1999, playback experiments; dataset 2: Quentin Gallot QG and Cassandre Depriester CD, 2021–2022, playback experiments; dataset 3: QG, 2022, predator visual model experiments). Olive colobus monkey groups were located within two large research areas (i.e., the north and south of the study site), separated from each other by around 2 km of dense forest. No Olive colobus monkey groups were observed between the two areas, suggesting that there was no interaction between them.

Olive colobus monkeys occupy small home ranges of about 0.56 km^2.^^29^ The species is endemic to West African rainforests and has been studied extensively to address questions about foraging behavior,^30^^,^^51^^,^^67^^,^^68^^,^^69^^,^^70^ morphology,^68^^,^^71^^,^^72^^,^^73^^,^^74^^,^^75^^,^^76^ anti-predation strategies^40^^,^^49^^,^^77^^,^^78^^,^^79^^,^^80^ and social and reproductive behavior.^29^^,^^31^^,^^32^^,^^40^^,^^41^^,^^46^^,^^51^^,^^68^^,^^77^^,^^81^^,^^82^ Like other monkeys in Taï National Park, the main predators of Olive colobus monkeys are crowned eagles, leopards and chimpanzees.^83^ These predators are common in Taï Forest with estimated densities of 0.1 leopards per km^2,^^84^ 0.4 eagles per km^2^^85^ and 1.8 chimpanzees per km^2.^^86^^,^^87^ Other dangers include falling trees and large branches,^88^ occasional lightning strikes, snake bites and human poaching.^89^

Olive colobus monkeys are highly cryptic, both behaviorally and morphologically, and remarkably successful in avoiding predation (leopard^90^; eagle^78^; chimpanzee^91^). Locating individuals is extremely difficult, even by very experienced observers, particularly because of their cryptic coloration and behavior and the fact that they spend the majority of their time in dense vegetation in the forest understory.^29^ One way to find them is by searching for noisy Diana monkey groups, the preferred association partner of Olive colobus monkeys.^29^^,^^40^

#### Playback experiments

Dataset 1: The first source of data were from recordings of unhabituated Olive colobus monkeys residing with Diana monkey groups that were exposed to playbacks of predator vocalizations (leopard growls, eagle shrieks). This first dataset was compiled by KZ between 1994 and 1999, comprising *N* = 24 trials (*N* = 18 trials with vocal responses) administered to *N* = 11 vocally responsive groups, some of which were tested with more than one stimulus (Table 1; also see Methods S1).

Dataset 2: The second source of data were also from unhabituated groups, comprising *N* = 201 trials (*N* = 91 trials with vocal responses) administered to *N* = 18 responsive groups between 2021 and 2022 (Table 1). These groups were exposed to leopard growls, eagle shrieks, and chimpanzee pant-hoots by QG and the sounds of falling trees by QG and CD.

For both datasets 1 and 2, we used recordings of leopard growls purchased from the British Library of Wildlife Sounds (BBC master tape number MM35; South African Broadcasting Corporation), whereas eagle and chimpanzee vocalizations and falling tree sounds were recorded directly in the study area. Some additional falling tree sounds were purchased from the BBC Sound Effects library (sounds 07002293, 07058030, 07058129). To avoid pseudo-replication, different versions of each stimulus type were produced (*N* = 4 eagle shrieks of 15 s, *N* = 4 leopard growls of 15 s, *N* = 2 chimpanzee pant-hoots of 15 s, and *N* = 7 falling tree sounds of 9 or 15 s) so that a particular group only contributed with one reaction per version. Sounds were broadcast with a NAGRA DSM speaker, typically positioned near the ground or in the canopy (up to 30 m: eagle and leopard playbacks only). We adjusted the speaker volume so that all stimuli were broadcast with a naturally sounding range (Table S8).

For dataset 1, the presence of an Olive colobus was noted after the end of the trial by visual or auditory detection. For dataset 2, trials were only initiated if there was evidence of Olive colobus monkeys before the trial, which allowed us to calculate response frequencies. Vocal and behavior responses from the monkeys were recorded up to 5 min after the start of each trial but usually vocal activity had ceased after 2–3 min, so we only considered the first 160 s of recording.

#### Predator model experiments

Dataset 3: We also recorded alarm calls given in response to visual predator models. Here, QG tested *N* = 8 unhabituated groups, resulting in *N* = 25 trials (10 trials with vocal responses, Table 1). Again, groups were located, monitored and recorded as before. We used both a leopard model, which consisted of an experimenter covering himself with leopard-patterned fabric mimicking the size, shape and posture of a leopard (Figure S5) and a crowned eagle model, which consisted of an experimenter hiding behind a camouflage net displaying a life-size 3D printed and hand-painted crowned eagle (Figure S5; see SWISSUbase: https://doi.org/10.60544/g1sx-4j76 for 3D print file). In both cases, the experimenter slowly approached a subject in the periphery of the group. All models were presented in motion to facilitate detection. After detection, the predator model remained in sight of the focal subject for a maximum of 1 min before slowly moving away and hiding behind a tree trunk. To address issues with pseudo-replication, we presented two different versions of each predator model (Figure S5) to monkey groups. We registered behavioral and vocal responses from the monkeys for up to 3 min after model detection. Vocal responses rarely lasted more than 160s, so we fixed the analysis window to 160s.

For all three datasets, we systematically searched the study area until a mixed species group was found, typically by hearing Diana monkey or red colobus monkey vocalizations. We then determined the group’s general behavior, species composition, location and variables related to visibility (i.e., illumination, vegetation density). We always tried to remain undetected by avoiding visual contact with the group, and monitored their behavior from a distance of about 25–75 m for at least 15 min prior to initiating a playback trial. Since we worked with unhabituated groups whose home ranges were unknown, to avoid retesting a group with the same stimulus more than once, we did not test any group in an area of 1 km around the location of the experiment for at least one week (Olive colobus mean home range = 0.56 km^2^, corresponding to an 850-metre diameter disc^29^), and we ensured that each stimulus was never presented more than once at the same location.

#### Equipment

CD and QG determined the location of monkey groups with a Garmin GPS RINO 655t and a Garmin GPS map 62s. KZ determined the location using a detailed map of the forest (Figure S6). QG played back sounds from a Samsung Galaxy XCover 4 (model SM-G390F, with media sound on maximum volume) connected to a Nagra Kudelski DSM-monitor loudspeaker (frequency response 60–15,000Hz ± 4 dB; Figure S7). CD played back sounds from an iPhone 5S (iOS 12.5.4) connected to an Alpha speaker (AER, The Acoustic People: frequency range 60–18,000 Hz). KZ played back sounds from a Sony WMD6C Professional Recorder connected to a Nagra Kudelski DSM-monitor loudspeaker. To simulate events as naturally and consistently as possible, stimulus amplitude was calibrated at 1 m from the speaker with sound level meters (QG: Standard ST-85C; CD: Decibel X app v9.4.0 on an iPhone 5S iOS 12.5.4; KZ: Radio Shack sound level meter 33–2050). KZ recorded vocal responses with Sony WMD6C or TCM5000EV recorders and Sennheiser ME88 or ME67 directional microphones (frequency response, 40–20,000Hz ± 2.5 dB). QG and CD recorded responses with a Marantz PMD 661 MKII recorder and a Sennheiser MKH 416 P48 directional microphone (frequency response, 40–20,000Hz ± 2.5 dB). 1994–99 recordings were digitized with Audacity software v2.1.0 (44.1 kHz sampling rate, 16 bits accuracy, WAV format;^58^) using TASCAM CD-A500 and Technics M280 devices.

### Quantification and statistical analysis

#### Call type repertoire

We extracted all calls from a total of *N* = 119 recordings (Table 1) in order to subject them to semi-automatic parameter extraction using Raven Pro software v1.6.1 (FFT size 2048, Hann window, hop size = 2 ms;^36^). To identify the main call types of the monkeys’ repertoire, we ran a Partitioning Around Medoids unsupervised clustering on all datasets (*N* = 1,246 calls), using the ‘pam’ function of the ‘cluster’ R package version 2.1.4.^62^ This analysis is less sensitive to outliers compared to a k-means method. We only considered two acoustic parameters: the duration and the maximum frequency of each call. A Partitioning Around Medoids model-based clustering requires the user to input the number of clusters, so we ran cluster solutions for a range from 1 to 10 clusters. We calculated the silhouette coefficient to assess the discreteness of cluster solutions using the ‘fviz_nbclust’ function of ‘factoextra’ R package v1.0.7^63^ and chose the cluster solution that had the highest silhouette coefficient. Silhouette coefficients provide a measure of how similar an object is relative to the established clusters. Values range from −1 to 1, and higher silhouette coefficients indicate a more appropriate clustering solution.^92^

#### The combinatoriality of call sequences

Calls were mostly produced as part of long call sequences. To define objectively a call sequence (i.e., a limit value to differentiate between inter-call intervals within sequences and inter-call intervals between sequences), we ran a kernel density estimation analysis via unsupervised model-based clustering method on inter-call interval data from all datasets, using the ‘mclust’ R package v6.1.1.^65^ To validate 2 components as the optimal number of modes to fit the inter-call interval distribution, we performed a bootstrap likelihood ratio test (*N* = 1,000 iterations), comparing successively models having between 1 and 5 modes, also using the ‘mclust’ R package.

To extract eventual rules in the sequence composition, we plotted a trie on all datasets (*N* = 284 sequences), using the ‘igraph’ R package v1.4.2.^64^

To explore the relation between external events and vocal production we tested a number of generalized linear mixed models (GLMM, see Table S9 for a detail description of each model). We built each model using the ‘glmmTMB’ function of the ‘glmmTMB’ R package v1.1.9.^60^ If complete or quasi-complete separation problem (i.e., when the outcome variable separates a predictor variable or a combination of predictor variables completely, in other words when one treatment combination has a response variance of zero or close to zero) was detected when trying to fit a model, we corrected the model by imposing independent zero-mean Gaussian priors with a standard deviation of 1 on all fixed effects, pushing variances away from zero (see^93^^,^^94^ for STAR Methods explanation).

We checked the model assumptions using functions from base R and the ‘DHARMa’ R package v0.4.6.^59^ Models were not under- or over-dispersed (DHARMa non-parametric dispersion test via SD of residual fitted vs. simulated: *p* > 0.05 for all models, Table S10), no influential outliers were detected (DHARMa bootstrapped outlier test: *p* > 0.05 for all models, Table S10), no zero-inflation (DHARMa zero-inflation test via comparison of observed vs. expected zeros: *p* > 0.05 for all models, Table S10). Visual inspection of the Q-Q plots confirmed the normality of the residuals (Kolmogorov-Smirnov test: *p* > 0.05 for all models, Table S10), and random factor is normally distributed in all models. We assessed model stability (i.e., minimum and maximum estimates) by comparing the full model estimates with those from models from which random effects were removed one at a time, using the ‘influence_mixed’ function of ‘glmmTMB’ R package v1.1.9. We reported robust 95% confidence intervals of model estimates which were derived by means of bootstraps (*N* = 1,000 iterations) with the ‘bootstrap_parameters’ function of ‘parameters’ R package v0.21.6.^66^

To assess overall significance of the fixed effects and avoid ‘cryptic multiple testing’,^95^ we used the ‘anova’ R function within base R to perform chi-squared likelihood ratio tests, contrasting each full model against a null model devoid of the fixed effects but including the random factor.^96^ To obtain individual *p*-values for all fixed effects and their interactions we compared the full model with a series of models in which each fixed effect was systematically dropped one at a time, using the ‘drop1’ function from base R. If an interaction between fixed effects were not significant, the model was refitted without this interaction. When post-hoc analyses were necessary, we conducted pairwise post-hoc comparisons between levels of statistically significant predictors by computing estimated marginal means for each model, using the ‘emmeans’ function of the ‘emmeans’ R package v1.8.5.^61^ For these comparisons, we included a Tukey honest significant difference adjustment to account for running multiple tests on the same data of the GLMM.^97^ In all performed tests, significance was tested at an alpha level of 0.05. All the statistical analyses were done using R software v4.3.1.^57^

To understand whether the production of specific sequence types (i.e., specific first call, overall pattern, or last bigram) during a trial depended on the playback stimulus type or the group location, we fitted three GLMMs (*N* = 104 trials, Table S9) with a Conway–Maxwell–Poisson error structure and log link function (GLMM1, GLMM5; under-dispersed count data) or a Poisson error structure and log link function (GLMM3). In all three models the response variable indicated the number of sequences produced per trial with one count for each sequence type (‘N_sequences’). In GLMM1, we focused on variations in the first call of the sequences produced, with the predictors being: the interaction between the sequence initiation (‘call_type’: A or B as first call of a sequence) and the playback stimulus (‘stimulus’: leopard, eagle or tree sounds), and the interaction between sequence initiation and group location (‘group_location’: north or south area of the study site). In GLMM3, we looked at variations in the overall pattern of sequences produced, with the predictors being: the interaction between the sequence pattern (‘pattern’: A, BA, A + BA or BA + A patterns) and the playback stimulus, and the interaction between sequence pattern and group location. In GLMM5, we focused on variations in the last bigram of sequences produced, with the predictors being: the interaction between the sequence termination (‘bigram type’: BA-gram or AA-gram as last calls of a sequence) and the playback stimulus, and the interaction between sequence termination and group location.

We also wanted to explore the effect of playback elevation on the quantity of the different sequence types produced during a trial. As the falling tree sounds were presented only near the ground, we fitted three new GLMMs excluding trials of falling tree playbacks (*N* = 64 trials, Table S9) with a Conway–Maxwell–Poisson error structure and log link function (GLMM2, GLMM6; under-dispersed count data) or a Poisson error structure and log link function (GLMM4). In all three models the response variable indicated the number of sequences produced per trial with one count for each sequence type. In GLMM2, we focused on variations in the first call of the sequences produced, with the predictors being: the interaction between the sequence initiation, the playback stimulus and the playback elevation (‘elevation’: down near the ground or up in a tree). In GLMM4, we looked at variations in the overall pattern of sequences produced, with the predictors being: the interaction between the sequence pattern, the playback stimulus, and the playback elevation. In GLMM6, we focused on variations in the last bigram of sequences produced, with the predictors being: the interaction between the sequence termination, the playback stimulus, and the playback elevation.

Because the samples of all models (GLMM1–6) were composed of different counts collected from the same trials, to avoid pseudo-replication, we included trial ID as a random factor in each model. We also added the log-transformed total number of sequences in a trial as an offset term in each model, allowing us to account for differences in vocal activity between trials (see Bolker^98^).

## Supporting citations

The following references appear in the supplemental information: ^56^.

## Supplemental information

Document S1. Figures S1–S7, Tables S1–S10, and Method S1
