# Supplementary material for: A non-human primate combinatorial system for long-distance communication
Source: iScience. 2024 Oct 15;27(11):111172. doi: 10.1016/j.isci.2024.111172 (PMC11570502; doi:10.1016/j.isci.2024.111172)
Supplement: Document S1. Figures S1–S7, Tables S1–S10, and Method S1 [file mmc1.pdf]

iScience, Volume *27*

Supplemental information

A non-human primate combinatorial system for long-distance communication

Quentin Gallot, Cassandre Depriester, Steven Moran, and Klaus Zuberbühler

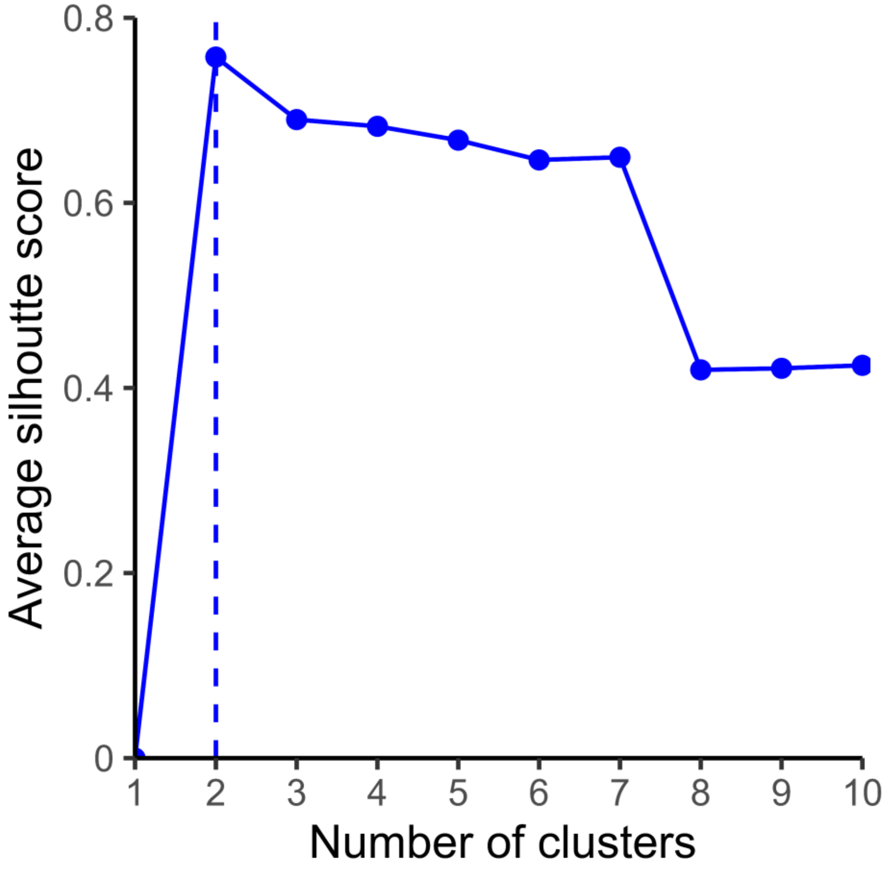

Figure S1. Optimal number of clusters based on average silhouette score, related to Figure 1.

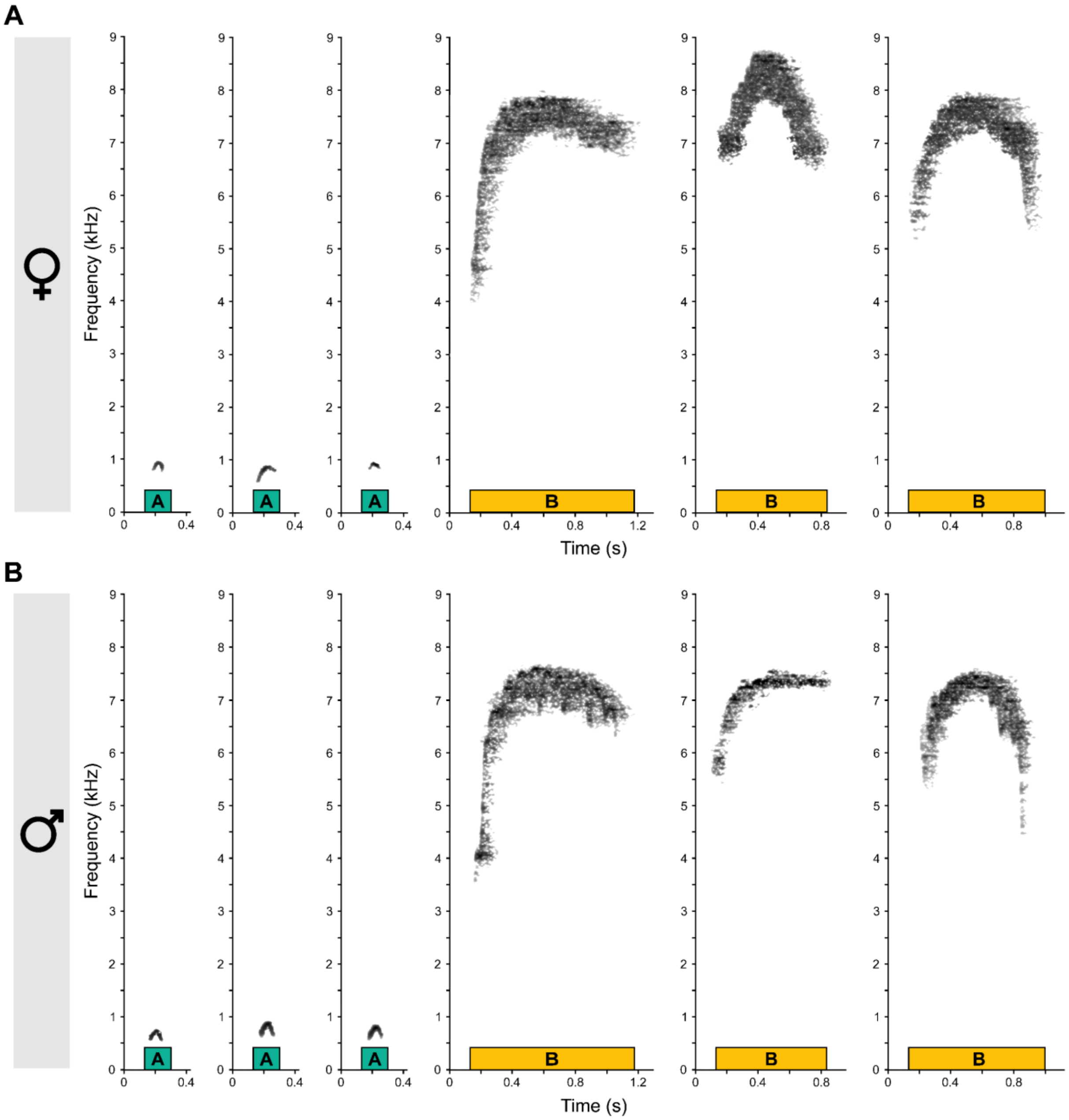

Figure S2. Spectrographic representations of two main Olive Colobus call types given by different adult females (A) and males (B) recorded from four different groups, related to Figure 1. Spectrograms were extracted from Raven Pro software v1.6.4, background noise removed with Adobe Photoshop software v23.0.0.

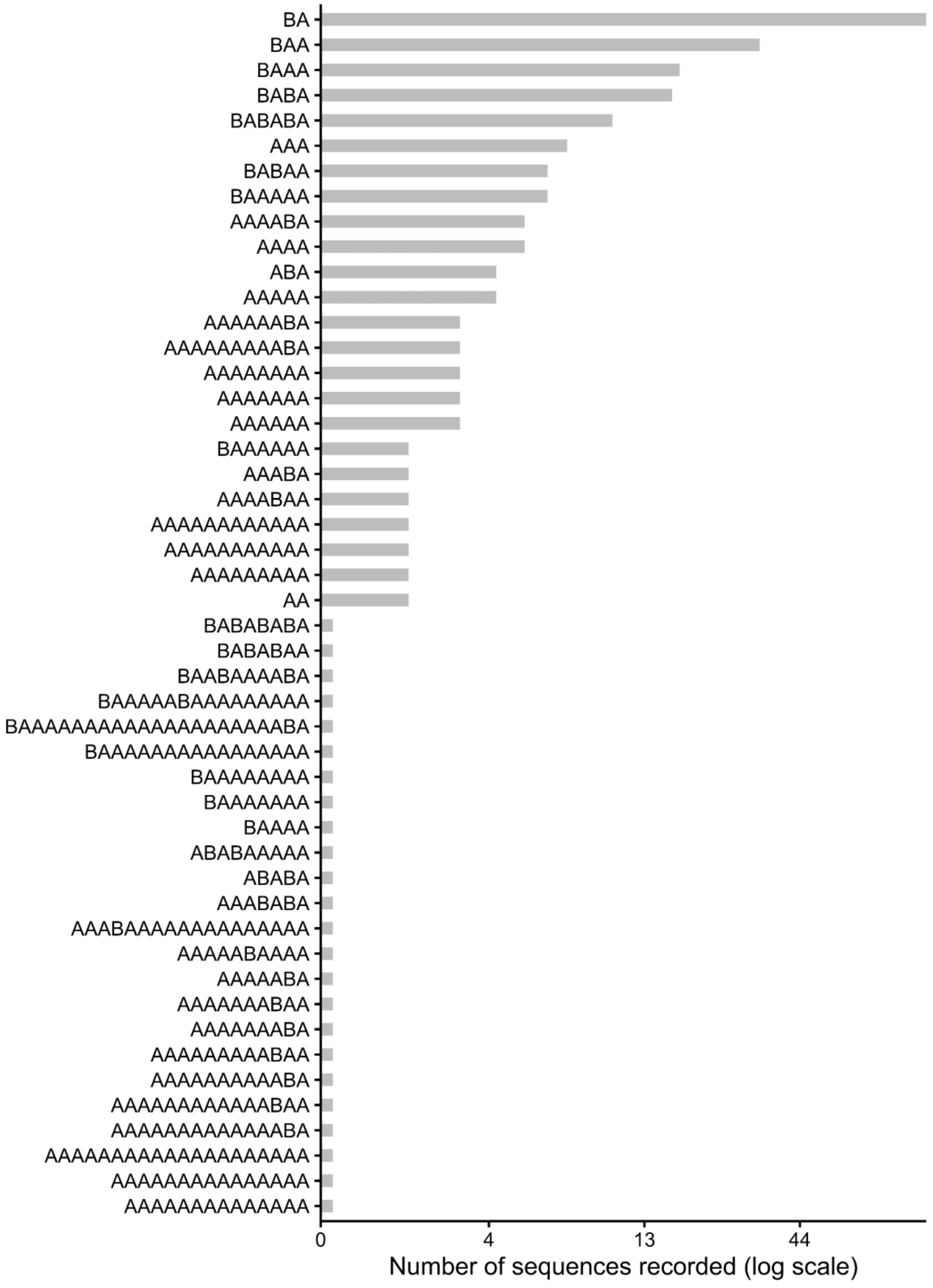

Figure S3. Ranked frequency of occurrences of Olive Colobus vocal sequences, related to Figure 3. Each line is a unique vocal sequence, with ‘A’ and ‘B’ representing Olive Colobus call types. The y-axis was log-transformed for ease of visualisation.

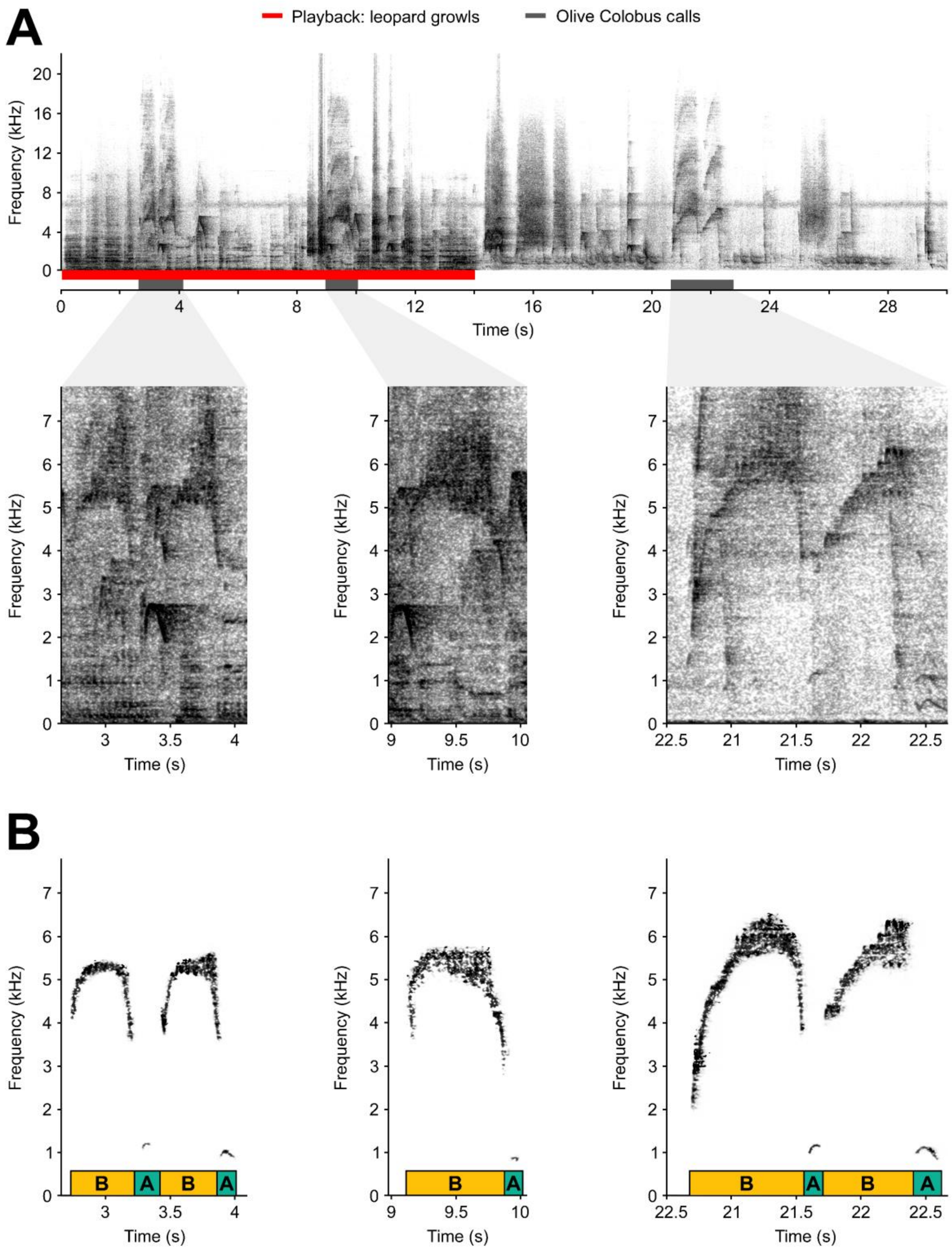

Figure S4. Spectrographic representation of (A) the first 30 seconds of a playback trial with (B) cleaned spectrograms of Olive Colobus sequences in response to leopard growls, related to Figure 4. Spectrograms were generated by Raven Pro software v1.6.4. Background noise was removed with Adobe Photoshop software v23.0.0.

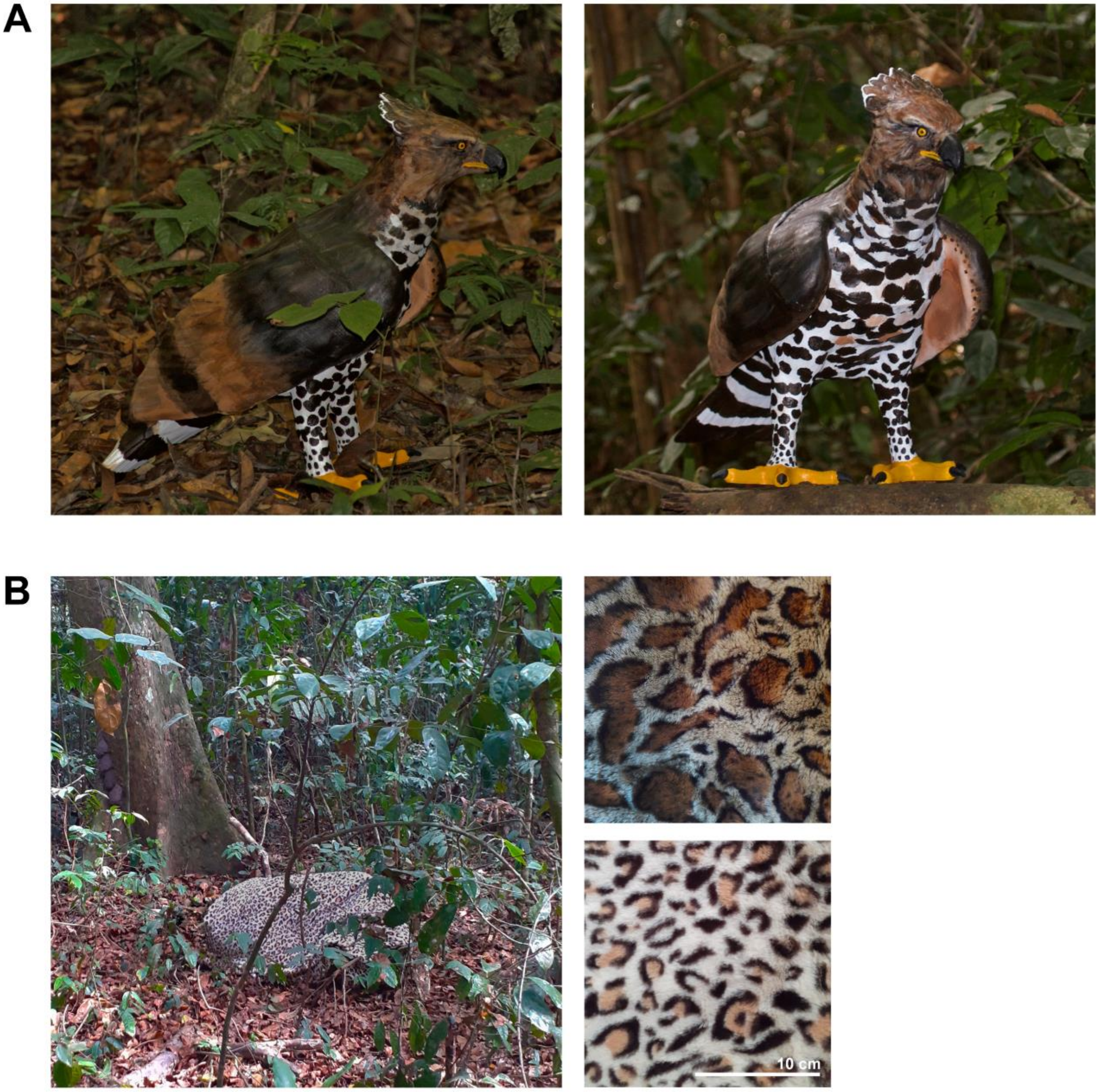

Figure S5. Lifesize animal models presented to unhabituated Olive Colobus groups, related to STAR Methods. (A) Two differently painted versions of a life-sized, anatomically-correct African crowned eagle. We 3D-printed the model using the UltiMaker Cura software v5.2.2 and the Anycubic Chiron 3D printer. (B) An experimenter on the ground with his head and body completely covered by one of two leopard fur patterned fabrics, mimicking the size, shape, and posture of a leopard.

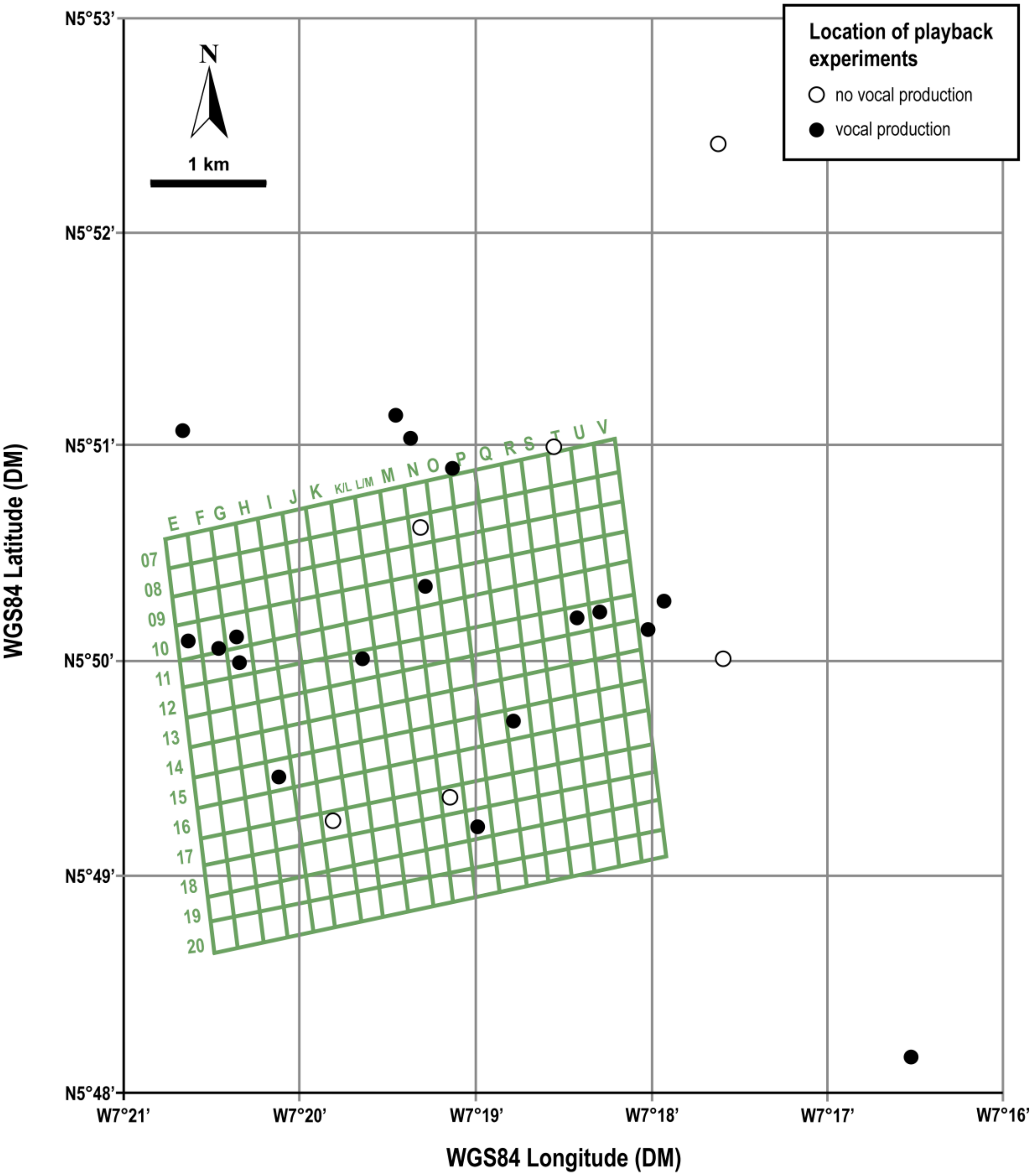

Figure S6. Original grid map used for the spatial coordinates of all playback experiments (*N* = 24 trials) by KZ between 1994 and 1999 in the Taï monkey project (Taï National Park, Ivory Coast), related to STAR Methods. Each data point represents the location of the focal Olive Colobus group tested during the trial. Its colour indicates Olive Colobus vocal production in response to the playback (white = no vocal production, black = vocal production of one individual).

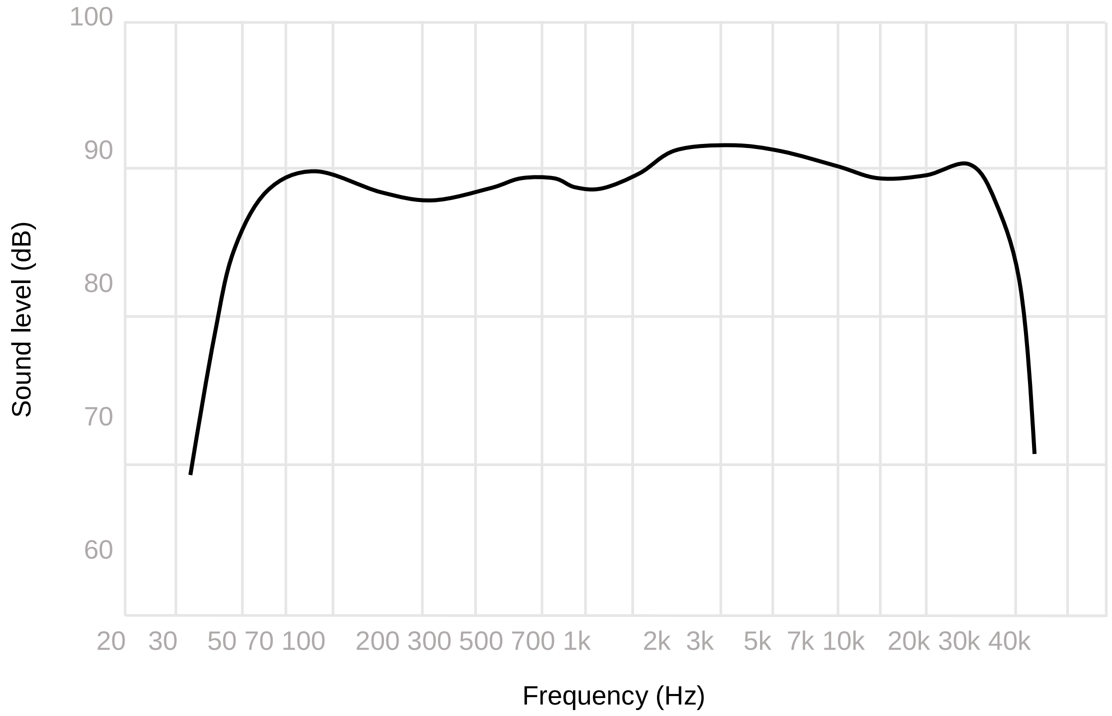

Figure S7. Nagra Kudelski DSM-monitor loudspeaker frequency response, related to STAR Methods. Official measurements released by NAGRA Kudelski: free field measurement with B&K condenser microphone for 1/10 power max on AC line. Sound level at 1 metre from the speaker.

Table S1. Call sequences unclassified, produced by Olive Colobus monkeys in response to different playback stimuli, related to Figure 4

Danger type	Playback stimulus	Call sequence [a]
eagle shrieks	E2	ABABAAAAA
	E2	AAAABAA
	E2	AAAAAAAAAAAABAA
falling tree sounds	TREE1	BAAAAABAAAAAAAAA
	TREE1	AAABAAAAAAAAAAAAAA
	TREE2	BAABAAAABA
	TREE2	BAAAAAAAAAAAAAAAAAAAABA
	TREE2_short	AAAAAAABAA
	TREE4	AAAABAA
	TREE4	AAAAABAAAA
	TREE5	AAAAAAAAABAA

[a] Each line represents a sequence, with ‘A’ and ‘B’ representing Olive Colobus call types.

Table S2. Pairwise post-hoc comparisons among playback stimulus types after refitting GLMM1 without non-significant predictor interactions, related to Figure 5. CI refers to the bootstrapped confidence interval (*N* = 1,000 iterations). Significant results are highlighted in bold.

Sequence initiation

GLMM1 refit formula: N_sequences ~ call_type * stimulus + group_location + offset(log(N_tot_sequences +1)) + (1 | trial_ID)

**Call_type**	**Stim. contrast**	**Estimate [a]**	**SE**	**95% CI**	**z**	**p [b]**
call A	*eagle - leopard*	2.207	0.285	[1.644, 2.847]	7.732	**<0.001**
	eagle - tree	1.127	0.221	[0.714, 1.566]	5.351	**<0.001**
	leopard - tree	-1.081	0.316	[-1.746, -0.471]	-3.424	**0.002**
call B	*eagle - leopard*	-2.158	0.413	[-3.247, -1.543]	-5.229	**<0.001**
	eagle - tree	-1.767	0.418	[-2.829, -1.096]	-4.228	**<0.001**
	leopard - tree	0.391	0.102	[0.200, 0.624]	3.852	**<0.001**

[a] Results are averaged over the levels of ‘group_location’

[b] P value adjustment: Tukey method for comparing a family of 3 estimates

Table S3. Results of GLMM2 exploring the effect of the interaction between playback stimulus type and playback elevation on the number of sequences starting with call A or call B, produced by an Olive Colobus during a trial (*N* = 62 trials), related to Table 2. The model was refitted without non-significant predictor interactions. CI refers to the bootstrapped confidence interval (*N* = 1,000 iterations), and min and max to minimum and maximum estimate from stability analysis. Significant results are highlighted in bold.

Sequence initiation

GLMM2 2nd refit formula [(a)] : N_sequences ~ call_type * stimulus + elevation + offset(log(N_tot_sequences +1)) + (1 | trial_ID)

Predictors	Estimate	SE	95% CI	min	max	χ^2	df	p
(Intercept)	-0.599	0.106	[-0.823, -0.429]	-0.629	-0.559	–	–	–
call_type [(c)]	–	–	–	–	–	–	–	[(b)]
call B	-1.821	0.416	[-2.920, -1.172]	-2.335	-1.709	–	–	–
stimulus [(d)]	–	–	–	–	–	–	–	[(b)]
leopard	-2.240	0.273	[-2.984, -1.740]	-2.540	-2.124	–	–	–
elevation [(e)]	–	–	–	–	–	2.438	1	0.119
up	-0.193	0.128	[-0.520, 0.044]	-0.235	-0.174	–	–	–
call_type: stimulus	–	–	–	–	–	118.612	1	**<0.001**
call B : leopard	4.373	0.488	[2.582, 5.720]	4.232	4.903	–	–	–

[(a)] Testing main effect of predictors after successively removing non-significant interaction from the model
GLMM2 *call_type : stimulus : elelvation*, χ^2 = 1.730, df = 1, *p* = 0.188
GLMM2 1st refit *call_type : elevation*, χ^2 = 0.025, df = 1, *p* = 0.875

[(b)] Not depicted because of limited interpretability

[(c)] Estimate refer to comparison with reference category '*call A*'

[(d)] Estimate refer to comparison with reference category '*eagle*'

[(e)] Estimate refer to comparison with reference category '*down*'

Table S4. Pairwise post-hoc comparisons among playback stimulus types after refitting GLMM3 without non-significant predictor interactions, related to Figure 6.
CI refers to the bootstrapped confidence interval (*N* = 1,000 iterations). Significant results are highlighted in bold.

Sequence pattern
GLMM3 refit formula: N_sequences ~ pattern * stimulus + group_location + offset(log(N_tot_sequences +1)) + (1 | trial_ID)

Pattern	Stim. contrast	Estimate [(a)]	SE	95% CI	z	p [(b)]
A	*eagle - leopard*	0.964	0.325	[0.078, 1.086]	2.965	**0.009**
	eagle - tree	0.323	0.313	[-0.448, 0.679]	1.032	0.557
	leopard - tree	-0.641	0.354	[-1.001, 0.195]	-1.810	0.166
BA	*eagle - leopard*	-1.539	0.329	[-1.899, -0.882]	-4.674	**<0.001**
	eagle - tree	0.538	0.439	[-0.008, 1.473]	1.226	0.438
	leopard - tree	2.076	0.347	[1.442, 2.652]	5.985	**<0.001**
A+BA	*eagle - leopard*	1.943	0.469	[0.776, 2.436]	4.139	**<0.001**
	eagle - tree	1.671	0.488	[0.446, 2.078]	3.421	**0.002**
	leopard - tree	-0.272	0.599	[-1.240, 0.552]	-0.455	0.892
BA+A	*eagle - leopard*	-0.479	0.429	[-0.700, 0.541]	-1.117	0.504
	eagle - tree	-1.720	0.404	[-1.841, -0.722]	-4.253	**<0.001**
	leopard - tree	-1.240	0.266	[-1.801, -0.760]	-4.663	**<0.001**

[(a)] Results are averaged over the levels of ‘group_location’

[(b)] P value adjustment: Tukey method for comparing a family of 3 estimates

Table S5. Results of GLMM4 exploring the effect of the interaction between playback stimulus type and playback elevation on the number of sequences for each sequence pattern, produced by an Olive Colobus during a trial (*N* = 62 trials), related to Table 3. The model was refitted without non-significant predictor interactions. CI refers to the bootstrapped confidence interval (*N* = 1,000 iterations), and min and max to minimum and maximum estimate from stability analysis. Significant results are highlighted in bold.

Sequence pattern

GLMM4 2nd refit formula [a] : N_sequences ~ pattern * stimulus + elevation + offset(log(N_tot_sequences +1)) + (1 | trial_ID)

Predictors	Estimate	SE	95% CI	min	max	χ^2	df	p
(Intercept)	-1.713	0.258	[-2.568, -1.497]	-1.801	-1.665	–	–	[b]
pattern [c]	–	–	–	–	–	–	–	[b]
BA	-0.303	0.383	[-0.487, 0.713]	-0.499	-0.182	–	–	–
A+BA	0.262	0.342	[-0.301, 1.072]	0.084	0.340	–	–	–
BA+A	-1.245	0.480	[-2.187, -0.304]	-1.322	-1.148	–	–	–
stimulus [d]	–	–	–	–	–	–	–	[b]
leopard	-0.968	0.330	[-1.076, 0.009]	-1.134	-0.904	–	–	–
elevation [e]	–	–	–	–	–	1.376	1	0.241
up	-0.244	0.208	[-0.653, 0.136]	-0.277	-0.233	–	–	–
pattern : stimulus	–	–	–	–	–	54.397	3	**<0.001**
BA : leopard	2.479	0.434	[1.234, 2.511]	2.382	2.658	–	–	–
A+BA : leopard	-1.056	0.523	[-1.973, -0.409]	-1.207	-0.902	–	–	–
BA+A : leopard	1.787	0.532	[0.745, 2.829]	1.535	1.943	–	–	–

[a] Testing main effect of predictors after successively removing non-significant interaction from the model
GLMM4 *pattern : stimulus : elelvation*, χ^2 = 2.980, df = 3, *p* = 0.395
GLMM4 1st refit *pattern : elevation*, χ^2 = 3.832, df = 3, *p* = 0.280

[b] Not depicted because of limited interpretability

[c] Estimates refer to comparison with reference category '*A*'

[d] Estimate refer to comparison with reference category '*eagle*'

[e] Estimate refer to comparison with reference category '*down*'

Table S6. Pairwise post-hoc comparisons among playback stimulus types after refitting GLMM5 without non-significant predictor interactions, related to Figure 7. CI refers to the bootstrapped confidence interval (*N* = 1,000 iterations). Significant results are highlighted in bold.

Sequence termiation

GLMM5 refit formula: N_sequences ~ bigram_type * stimulus + group_location + offset(log(N_tot_sequences +1)) + (1 | trial_ID)

Bigram_type	Stim. contrast	Estimate [(a)]	SE	95% CI	z	p [(b)]
AA-gram	*eagle - leopard*	0.527	0.243	[0.045, 0.999]	2.170	0.076
	eagle - tree	-0.624	0.213	[-1.031, -0.215]	-2.924	**0.010**
	leopard - tree	-1.151	0.175	[-1.505, -0.819]	-6.568	**<0.001**
BA-gram	*eagle - leopard*	-0.656	0.194	[-1.114, -0.316]	-3.374	**0.002**
	eagle - tree	1.084	0.331	[0.538, 1.833]	3.488	**0.001**
	leopard - tree	1.740	0.263	[1.295, 2.331]	6.625	**<0.001**

[(a)] Results are averaged over the levels of ‘group_location’

[(b)] P value adjustment: Tukey method for comparing a family of 3 estimates

Table S7. Results of GLMM6 exploring the effect of the interaction between playback stimulus type and playback elevation on the number of sequences ending with an 'AA-gram' or a 'BA-gram', produced by an Olive Colobus during a trial (N = 62 trials), related to Table 4. The model was refitted without non-significant predictor interactions. CI refers to the bootstrapped confidence interval (*N* = 1,000 iterations), and min and max to minimum and maximum estimate from stability analysis. Significant results are highlighted in bold.

Sequence termination

GLMM6 2nd refit formula [(a)] : N_sequences ~ bigram_type * stimulus + elevation + offset(log(N_tot_sequences +1)) + (1 | trial_ID)

Predictors	Estimate	SE	95% CI	min	max	χ^2	df	p
(Intercept)	-1.178	0.206	[-1.675, -0.826]	-1.242	-1.084	–	–	–
bigram_type [(c)]	–	–	–	–	–	–	–	[(b)]
BA-gram	0.073	0.283	[-0.472, 0.648]	-0.173	0.188	–	–	–
stimulus [(d)]	–	–	–	–	–	–	–	[(b)]
leopard	-0.545	0.261	[-1.020, 0.031]	-0.706	-0.478	–	–	–
elevation [(e)]	–	–	–	–	–	1.686	1	0.194
up	-0.228	0.182	[-0.638, 0.100]	-0.271	-0.209	–	–	–
bigram_type : stim.	–	–	–	–	–	12.414	1	**<0.001**
BA-gram : leo.	1.209	0.336	[0.491, 1.872]	1.093	1.448	–	–	–

[(a)] Testing main effect of predictors after removing non-significant interaction from the model
GLMM6 *bigram_type : stimulus : elevation*, χ^2 = 0.696, df = 1, *p* = 0.404
GLMM6 1st refit *bigram_type : elevation*, χ^2 = 1.917, df = 1, *p* = 0.166

[(b)] Not depicted because of limited interpretability

[(c)] Estimate refer to comparison with reference category '*AA-gram*'

[(d)] Estimate refer to comparison with reference category '*eagle*'

[(e)] Estimate refer to comparison with reference category '*down*'

Table S8. Amplitude measurements of all sound stimulus used during playback experiments, related to STAR Methods. All the stimuli used by KZ were played with a naturally sounding range with a maximum amplitude at 100 cm of the speaker between 88 and 100 dB (leopard growls: 88 to 92 dB, and eagle shrieks: 92 to 100 dB).

			At 50cm of the speaker		At 100cm of the speaker			
**Stimulus category**	**Stimulus**	**Experi-menter ***	**Average amplitude (dB)**	**Maximum amplitude (dB)**	**Average amplitude (dB)**	**Maximum amplitude (dB)**	**Nagra speaker 'sensitivity'**	**Alpha speaker 'gain'**
Chimpanzee pant-hoots	C(PH)1	QG	100	103.7	96.5	100.4	-21	NA
	C(PH)2	QG	101	102.5	96	101.7	-21	NA
Eagle shrieks	E1	QG	94.5	99.7	91	92.5	-12	NA
	E2	QG	96.5	98.7	92.5	93.9	-30	NA
	E3	QG	97	99.0	92	93.6	-30	NA
Leopard growls	L1	QG	91.5	101.8	90	96.1	-35	NA
	L2	QG	93.5	102.2	91	95.4	-35	NA
	L3	QG	93	102.4	88.5	90.1	-30	NA
Falling tree sounds	TREE1	QG	97	101.7	91	95.4	-40	NA
	TREE1_short	CD	93.2	101.5	89.9	97.3	NA	2.75
	TREE2	QG	96	100.4	92.5	97.1	-21	NA
	TREE2_short	CD	86.9	100.5	83	96.9	NA	3.5
	TREE4	CD	91.6	101.9	86.8	97.6	NA	4.5
	TREE5	CD	92.4	99.9	88.8	96.7	NA	3.5
	TREE6	CD	92	101.2	87.4	97.3	NA	4

*QG: Quentin Gallot / CD: Cassandre Depriester.

Table S9. Structure of the generalized linear mixed models (GLMMs) used in this study, related to STAR Methods.

GLMM	Response variable	Test variabes	Offset term [(a)]
GLMM1	N_sequences	call_type * (stimulus + group_location)	log(N_tot +1)
refit	N_sequences	call_type * stimulus + group_location	log(N_tot +1)
GLMM2	N_sequences	call_type * stimulus * elevation	log(N_tot +1)
refit	N_sequences	call_type * (stimulus + elevation)	log(N_tot +1)
refit2	N_sequences	call_type * stimulus + elevation	log(N_tot +1)
GLMM3	N_sequences	pattern * (stimulus + group_location)	log(N_tot +1)
refit	N_sequences	pattern * stimulus + group_location	log(N_tot +1)
GLMM4	N_sequences	pattern * stimulus * elevation	log(N_tot +1)
refit	N_sequences	pattern * (stimulus + elevation)	log(N_tot +1)
refit2	N_sequences	pattern * stimulus + elevation	log(N_tot +1)
GLMM5	N_sequences	bigram_type * (stimulus + group_location)	log(N_tot +1)
refit	N_sequences	bigram_type * stimulus + group_location	log(N_tot +1)
GLMM6	N_sequences	bigram_type * stimulus * elevation	log(N_tot +1)
refit	N_sequences	bigram_type * (stimulus + elevation)	log(N_tot +1)
refit2	N_sequences	bigram_type * stimulus + elevation	log(N_tot +1)

GLMM	Random factor	Error distribution	Fixed.ef. priors	N observations	N of trials
GLMM1	trial_ID	compois(link=log)	–	208	104
refit	trial_ID	compois(link=log)	–	208	104
GLMM2	trial_ID	compois(link=log)	normal(0,1)	124	62
refit	trial_ID	compois(link=log)	–	124	62
refit2	trial_ID	compois(link=log)	–	124	62
GLMM3	trial_ID	poisson(link=log)	normal(0,1)	416	104
refit	trial_ID	poisson(link=log)	normal(0,1)	416	104
GLMM4	trial_ID	poisson(link=log)	normal(0,1)	248	62
refit	trial_ID	poisson(link=log)	normal(0,1)	248	62
refit2	trial_ID	poisson(link=log)	normal(0,1)	248	62
GLMM5	trial_ID	compois(link=log)	–	208	104
refit	trial_ID	compois(link=log)	–	208	104
GLMM6	trial_ID	compois(link=log)	normal(0,1)	124	62
refit	trial_ID	compois(link=log)	normal(0,1)	124	62
refit2	trial_ID	compois(link=log)	–	124	62

[(a)] During some trials the Olive Colobus vocal response consisted of only single calls, resulting in 'N_tot' of 0. 1 was added to all 'N_tot' count to be able to log-transformed this offset term.

Table S10. Checks on GLMMs' assumptions, related to STAR Methods.

	Dispersion [(a)]		Residual's normality [(b)]		Outliers [(c)]	Zero-inflation [(d)]	
**GLMM**	**disp.**	**p**	**D**	**p**	**p**	**ratio Obs./Sim.**	**p**
GLMM1	1.028	0.744	0.063	0.374	0.162	1.022	0.824
refit	1.000	0.896	0.070	0.267	0.201	1.022	0.800
GLMM2	0.965	0.992	0.090	0.267	0.145	1.077	0.488
refit	1.007	0.848	0.090	0.269	0.101	0.985	0.936
refit2	1.007	0.952	0.076	0.470	0.120	0.979	0.936
GLMM3	0.802	0.136	0.050	0.259	0.581	1.023	0.560
refit	0.810	0.168	0.051	0.230	0.604	1.023	0.568
GLMM4	0.757	0.144	0.038	0.857	0.944	1.016	0.640
refit	0.730	0.104	0;047	0.657	0.760	1.003	0.999
refit2	0.781	0.208	0.044	0.735	0.865	1.020	0.680
GLMM5	0.972	0.872	0.073	0.213	0.991	1.101	0.232
refit	0.967	0.816	0.085	0.099	0.846	1.102	0.216
GLMM6	0.971	0.968	0.047	0.949	0.988	1.101	0.400
refit	0.953	0.792	0.062	0.728	1.000	1.097	0.384
refit	0.947	0.864	0.097	0.193	1.000	1.092	0.432

Statistical tests from DHARMa package:

(a) DHARMa non-parametric dispersion test via SD of residual fitted vs. simulated

(b) Kolmogorov–Smirnov test for normality

(c) DHARMa bootstrapped outlier test (*N* = 1,000 iterations)

(d) DHARMa zero-inflation test via comparison of observed vs. expected zeros

Methods S1. KZ protocol details from dataset 1, related to STAR Methods.

The playback experiments were designed using a prime-probe technique. In each experiment, a Diana monkey group heard two playback stimuli, separated by 5 min of silence. Across conditions, prime and probe stimuli varied with respect to their acoustic and referential (or semantic) resemblance (see for a more detailed discussion of the prime–probe paradigm in Zuberbühler et al. 1999, 2000). Recordings of the subjects' vocal behaviour began with the first playback stimulus and lasted for 15 min. As KZ's data collection was not designed to study Olive Colobus vocal production, QG screen the dataset to keep only independent trials. First, each experiment was divided in two separated recordings corresponding to two dependent trials with the stimulus order (i.e., prime or probe) added. Only the trials with leopard growls or eagle shrieks stimuli were investigated. Second, to avoid ambiguity in the interpretation of Olive Colobus responses, only the prime trails were kept in the dataset. Third, to keep trials independent, we considered the addition of two parameters: the home range of the Olive Colobus species (approximately 0.56 km^2, corresponding to an 850-metre diameter disc; McGraw et al. 2007) and the inaccuracy of GPS coordinates from the Taï forest grid (Figure S6; 250 metres = maximum size of the diagonal of a cell). We therefore only considered trials if GPS points of the respective experiments were located at least 1 km apart.
